# Synergistic Activation of HIV-1 Expression by Deacetylase Inhibitors and Prostratin: Implications for Treatment of Latent Infection

**DOI:** 10.1371/journal.pone.0006093

**Published:** 2009-06-30

**Authors:** Sophie Reuse, Miriam Calao, Kabamba Kabeya, Allan Guiguen, Jean-Stéphane Gatot, Vincent Quivy, Caroline Vanhulle, Aurélia Lamine, Dolores Vaira, Dominique Demonte, Valérie Martinelli, Emmanuelle Veithen, Thomas Cherrier, Véronique Avettand, Solène Poutrel, Jacques Piette, Yvan de Launoit, Michel Moutschen, Arsène Burny, Christine Rouzioux, Stéphane De Wit, Georges Herbein, Olivier Rohr, Yves Collette, Olivier Lambotte, Nathan Clumeck, Carine Van Lint

**Affiliations:** 1 Laboratory of Molecular Virology, Institut de Biologie et de Médecine Moléculaires (IBMM), Université Libre de Bruxelles (ULB), Gosselies, Belgium; 2 Service des Maladies Infectieuses, CHU St-Pierre, Université Libre de Bruxelles (ULB), Bruxelles, Belgium; 3 Faculté de Médecine Paris-Sud, INSERM U802, Bicêtre, France; 4 AIDS Reference Center, University of Liege (ULg), Liège, Belgium; 5 Virology Institute, INSERM U575, Strasbourg, France; 6 Service de Virologie, EA3620, Université Paris-Descartes, AP-HP, Hôpital Necker-Enfants-Malades, Paris, France; 7 Laboratory of Virology and Immunology, GIGA-R, University of Liege (ULg), Liège, Belgium; 8 Institut de Biologie de Lille, Institut Pasteur de Lille, UMR 8117 CNRS, BP447, Université de Lille 1, Lille, France; 9 Department of Virology, EA3186, IFR133, Franche-Comte University, Hôpital Saint-Jacques, Besançon, France; 10 Centre de Recherche en Cancérologie de Marseille, INSERM UMR 599, Marseille, France; Institut Pasteur Korea, Republic of Korea

## Abstract

The persistence of transcriptionally silent but replication-competent HIV-1 reservoirs in Highly Active Anti-Retroviral Therapy (HAART)-treated infected individuals, represents a major hurdle to virus eradication. Activation of HIV-1 gene expression in these cells together with an efficient HAART has been proposed as an adjuvant therapy aimed at decreasing the pool of latent viral reservoirs. Using the latently-infected U1 monocytic cell line and latently-infected J-Lat T-cell clones, we here demonstrated a strong synergistic activation of HIV-1 production by clinically used histone deacetylase inhibitors (HDACIs) combined with prostratin, a non-tumor-promoting nuclear factor (NF)- κB inducer. In J-Lat cells, we showed that this synergism was due, at least partially, to the synergistic recruitment of unresponsive cells into the expressing cell population. A combination of prostratin+HDACI synergistically activated the 5′ Long Terminal Repeat (5'LTR) from HIV-1 Major group subtypes representing the most prevalent viral genetic forms, as shown by transient transfection reporter assays. Mechanistically, HDACIs increased prostratin-induced DNA-binding activity of nuclear NF-κB and degradation of cytoplasmic NF-κB inhibitor, IκBα . Moreover, the combined treatment prostratin+HDACI caused a more pronounced nucleosomal remodeling in the U1 viral promoter region than the treatments with the compounds alone. This more pronounced remodeling correlated with a synergistic reactivation of HIV-1 transcription following the combined treatment prostratin+HDACI, as demonstrated by measuring recruitment of RNA polymerase II to the 5'LTR and both initiated and elongated transcripts. The physiological relevance of the prostratin+HDACI synergism was shown in CD8^+^-depleted peripheral blood mononuclear cells from HAART-treated patients with undetectable viral load. Moreover, this combined treatment reactivated viral replication in resting CD4^+^ T cells isolated from similar patients. Our results suggest that combinations of different kinds of proviral activators may have important implications for reducing the size of latent HIV-1 reservoirs in HAART-treated patients.

## Introduction

HIV-1 infection can be treated effectively in many patients in the developed world, using combinations of antiretroviral therapeutics, called Highly Active Anti-Retroviral Therapy (HAART). However, despite prolonged treatment with HAART, the persistence of HIV-1 reservoirs harboring transcriptionally silent but replication-competent proviruses represents the major hurdle to virus eradication. These latently-infected cells are a permanent source for virus reactivation and lead to a rebound of the viral load after interruption of HAART. Therefore, current anti-HIV-1 research efforts are increasingly focused on strategies aimed at reducing the size of these persistent reservoirs of latent HIV-1 by forcing viral gene expression. This kind of strategy would allow latently-infected cells to die from viral cytopathic effects or host cytolytic effector mechanisms following viral reactivation, while the antiretroviral therapy would prevent spreading of the infection by the neosynthetized virus [1], [2].

Acetylation level of histone and non-histone proteins, controlled by deacetylases (HDACs) and acetyltransferases (HATs), is a key element regulating HIV-1 transcription. In agreement, we have previously reported that treatment of latently HIV-1-infected cell lines with HDAC inhibitors (HDACIs) induces viral transcription and remodeling of the repressive nucleosome nuc-1, located immediately after the HIV-1 transcription start site under latency conditions [3], [4]. Similar results were observed in transiently or stably transfected HIV-1 Long Terminal Repeat (LTR) reporter constructs [5], [6], [7], and on *in vitro* chromatin-reconstituted HIV-1 templates [8], [9]. Based on these observations, administration of HDACIs together with efficient HAART has been proposed as an inductive adjuvant therapy for the decay of latent reservoirs [10], [11], [12], [13]. The Margolis group has reported that VPA (valproic acid), in the presence of IL-2, induces rescue of replication-competent HIV-1 from purified resting CD4^+^ T cells obtained from HAART-treated patients with undetectable viral load [14]. Later, in a clinical trial performed by same group, four patients receiving HAART and the viral entry inhibitor enfuvirtide were given VPA for three months, and a modest but significant decrease in the frequency of latently-infected cells was noted in three of the four patients [15]. However, given that at least two studies have demonstrated that intensification of anti-HIV therapy decreases the half-life of this population [16], [17], it is unclear whether VPA or intensification of HAART with enfuvirtide was the critical factor for the decay of the latent reservoir. Recent reports have failed to show a decay of resting CD4^+^ T cell infection in patients who were prescribed VPA for clinical reasons while receiving standard HAART [18], [19], [20]. These results led to question the therapeutic potential of VPA, at least when used alone, to reduce the size of the latent HIV-1 reservoirs.

We have previously demonstrated a strong synergistic activation of HIV-1 promoter activity by the HDACI trichostatin A (TSA) and the NF-κB inducer TNFα in the postintegration latency model cell line U1 [3], [21], suggesting that combinations of two independent factors (NF-κB and chromatin) involved in HIV-1 reactivation from latency might be potent tools to decrease the pool of latently-infected reservoirs. However, because of their toxicity, the therapeutic use of TNFα and TSA is not possible. Here, we investigated the HIV-1 reactivating potential of a treatment combining HDACIs used in human clinical trials or therapies (such as VPA and suberoylanilide hydroxamic acid [SAHA]) and an NF-κB inducer with no tumor-promoting effects, prostratin. This compound is an inducer of protein kinase C activity which stimulates HIV-1 expression in latently-infected lymphoid and myeloid cell lines and primary cells [22], [23], [24], [25], [26], [27], [28], [29] with minimal effects on the immune system [26] and causing minimal cell cycle progression [26], [29]. Moreover, prostratin also inhibits *de novo* HIV-1 infection via posttranscriptional downregulation of the cellular HIV-1 receptors CD4 and CXCR4 (CXC chemokine receptor 4) [24], [27], [30], [31], [32]. Therefore, the antimitogenic property of prostratin coupled with its dual activity on HIV-1 infection (inhibition of viral infection and upregulation of latent provirus expression), its relatively non-toxic behavior and its potential widespread effect on different HIV-1 reservoirs, make this compound a good candidate for viral purging. Although the suitability of prostratin for use in humans is unknown since preclinical testing in compliance with Food and Drug Administration regulations is reportedly still underway [12], [33], preliminary pharmacokinetic studies are encouraging as they show that 5 of 5 mice survived with no obvious effects at 100 µmol/Kg intragastric dose of prostratin with plasma concentrations reaching up to 1.42 µmol/L [24]. These results are not surprising as plant extracts of *Homalanthus nutans* (a source of prostratin) have already been used by the Samoan healers to treat individuals with certain medical conditions such as hepatitis [34], [35].

Here, we demonstrated that a combination of prostratin+VPA or prostratin+SAHA reactivated more efficiently than each compound alone HIV-1 production both in several latently-infected cell lines (U1 and J-Lat clones) and in CD8^+^-depleted peripheral blood mononuclear cells (PBMCs) isolated from HIV-1-infected patients receiving HAART and with undetectable viral load. Mechanistically, HDACI increased prostratin-induced NF-κB activation and potentiated nuc-1 remodeling. Moreover, prostratin+HDACI combined treatment caused a synergistic activation of HIV-1 transcriptional initiation and elongation. Our results constitute a proof-of-concept for the coadministration of two different types of therapeutically promising HIV-1 inducers (one acting on the NF-κB pathway and the other acting on the protein acetylation status) together with HAART as a therapeutic perspective to decrease the pool of latent HIV-1 reservoirs.

## Results

### Synergistic activation of HIV-1 production by prostratin and clinically used HDACIs

HDACIs present several advantages for HIV-1 purging strategies. They do not induce proliferation or activation of T cells [36], [37], act in a broad spectrum of cell lines and potently repress CXCR4 chemokine receptor expression and function [38]. Moreover, whereas TSA has a high toxicity and a costly and poorly efficient production [39], several HDACIs are safely administered for other diseases [40], [41], [42], [43], [44], [45] or tested in clinical trials as anticancer drugs [46]. HDACIs are specific for class I and II or class III HDACs and have been subdivided into four distinct groups based on their structural characteristics: hydroxamic acids; short-chain fatty acids; synthetic benzamide derivatives; cyclic tetrapeptides/epoxides [39], [46], [47].

**Figure 1:**
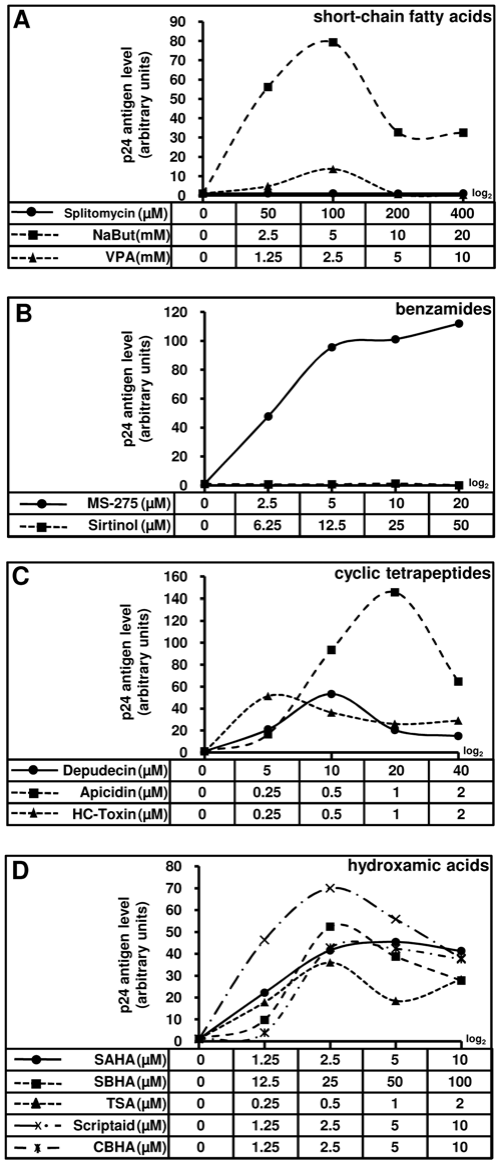
Dose-dependent effects of various HDACIs on HIV-1 production. U1 cells were mock-treated or treated with HDACIs: short-chain fatty acids (A), benzamides (B), cyclic tetrapeptides (C), hydroxamic acids (D). At 24 h posttreatment, viral production was estimated by measuring CA-p24 antigen concentration in culture supernatants. The mock-treated value was arbitrarily set at a value of 1. Each point is the mean from three separate experiments performed in triplicate. SE are intentionally not represented on the graph for clarity reasons.

For various HDACIs, we determined an optimal concentration in terms of both their cellular toxicity and their HIV-1 reactivation potential by measuring cell viability (Figure S1 and Text S1) and induction of p24 production in HDACI-treated U1 cells (Figure 1). The latently HIV-1-infected monocytic cell line U1 is one of the most studied cellular models of postintegration latency. All the inhibitors of class I and II HDACs we tested increased core antigen levels (p24) in the U1 cellular supernatants, independently of the structural group the HDACIs belong to (Figure 1A, 1B, 1C and 1D). In the short-chain fatty acid group, VPA was a weaker inducer of HIV-1 p24 production than NaBut (sodium butyrate) (Figure 1A). MS-275 induced viral p24 antigen production 112-fold at a concentration of 20 µM (Figure 1B). Among the cyclic tetrapeptide group, Apicidin at 1 µM exhibited the highest reactivation of viral production (Figure 1C). All HDACIs from the hydroxamic acid group were more efficient than TSA to induce HIV-1 p24 antigen production (Figure 1D). Splitomycin and Sirtinol, which are specific inhibitors of class III HDACs, had no effect on p24 production (Figure 1A and 1B, respectively).

**Figure 2:**
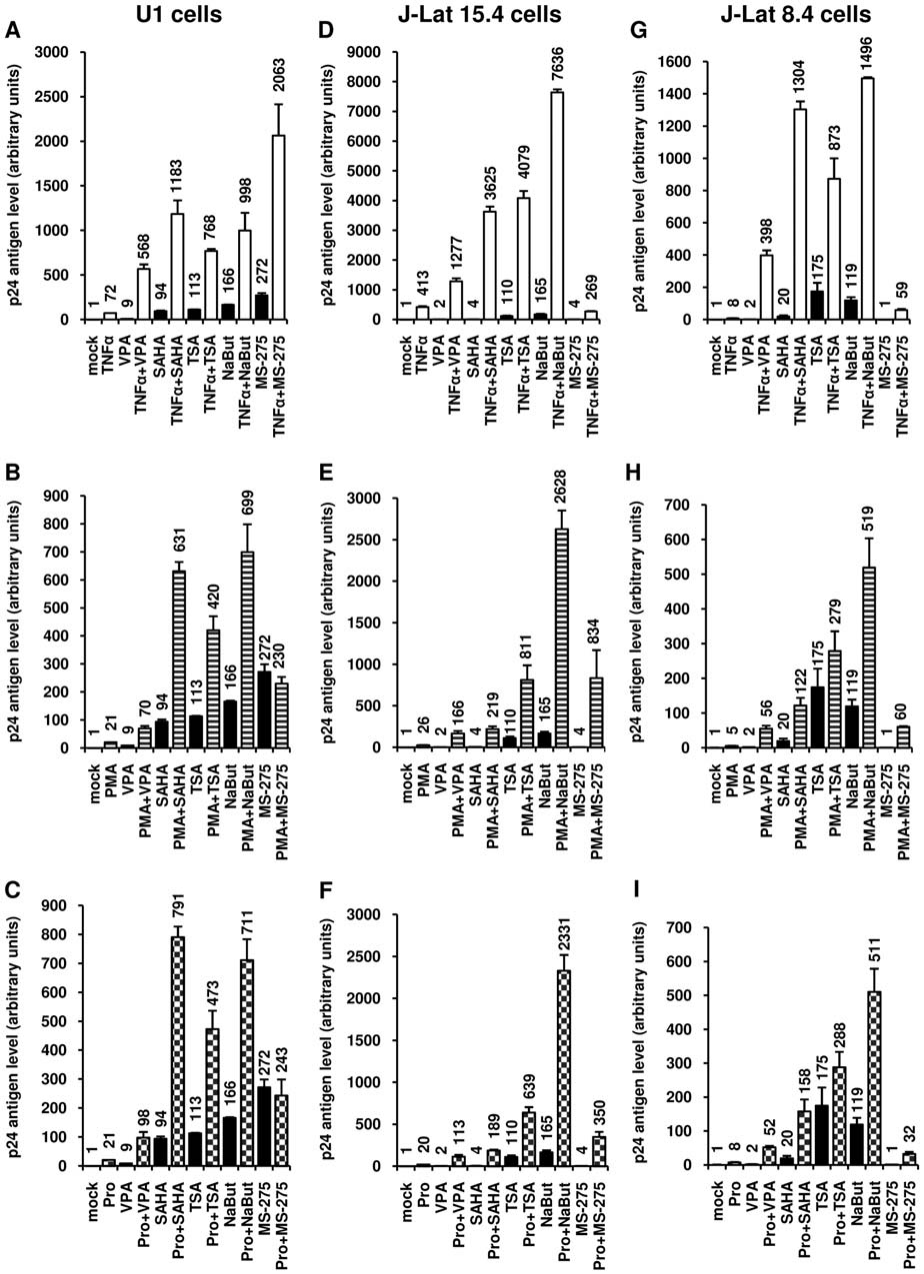
Synergistic activation of HIV-1 production by prostratin and clinically used HDACIs. U1 (panels A, B and C), J-Lat 15.4 (panels D, E and F) or J-Lat 8.4 (panels G, H and I) cells were mock-treated or treated with TNFα (10 ng/ml) (panels A, D and G), PMA (20 nM) (panels B, E and H), prostratin (5 µM) (panels C, F and I) alone or in combination with different HDACIs [VPA (2.5 mM), SAHA (2.5 µM), TSA (500 nM), NaBut (5 mM) or MS-275 (5 µM)]. At 24 h posttreatment, viral production was estimated by measuring CA-p24 antigen concentration in culture supernatants. The mock-treated value was arbitrarily set at a value of 1. Each value is the mean±SE from two (for the J-Lat 15.4 and 8.4 cell lines) or three (for the U1 cell lines) separate experiments performed in triplicate.

Among the HDACIs tested, VPA, NaBut and SAHA are already in clinical use to reduce epileptic seizures [43], to treat sickle cell anemia and beta-thalassemia [41] and for the treatment of cutaneous T-cell lymphoma [42], [48], [49], respectively, whereas others, such as MS-275 [50], are in clinical trials. We tested whether these HDACIs, already used in human therapy and thus promising for AIDS treatment, synergistically reactivated HIV-1 p24 antigen production in U1 cells when combined with TNFα, the tumor-promoting phorbol ester PMA (phorbol myristate acetate) or the non-tumor-promoting phorbol ester prostratin (Figure 2A, 2B and 2C). Two activators synergize when their combination produces a level of activation that is greater than the sum of the effects produced with the individual activators. HDACI concentrations were chosen according to our reactivation results (Figure 1) and our cytotoxicity results (Figure S1 and Text S1). HIV-1 p24 antigen production was synergistically activated by each TNFα+HDACI cotreatment (Figure 2A). Similarly, we observed important synergistic activations of virus production with each combination of the phorbol esters PMA (Figure 2B) or prostratin (Figure 2C) with an HDACI, except for MS-275.

In order to extend the results obtained in the U1 monocytic latency model cell line, we decided to confirm our results in the Jurkat CD4^+^ T-cell-based model of HIV-1 postintegration latency termed J-Lat [51]. J-Lats are T-cell clones containing a single, full-length integrated HIV-1 provirus with the green fluorescent protein (GFP) open reading frame in place of the *nef* gene, thus permitting epifluorescence monitoring of viral transcriptional activity. Under basal conditions, little or no GFP expression is detected and transcriptional activation of the latent provirus leads to GFP expression, which can be detected at the single-cell level by flow cytometry. In contrast to several other HIV-1 postintegration model cell lines (including the U1 cell line [52]), the J-Lat clones do not contain mutations in the HIV-1 *tat* (*trans*-activator of transcription) gene or TAR element (*trans*-activation response element). We observed important synergistic activations of viral production in the two J-Lat clones tested, the J-Lat 15.4 (Figure 2D, 2E and 2F) and the J-Lat 8.4 (Figure 2G, 2H and 2I) cells, with each combination of TNFα (Figure 2D and 2G), PMA (Figure 2E and 2H) or prostratin (Figure 2F and 2I) with an HDACI. Of note, both PMA and prostratin synergistically activated virus production with MS-275 in the J-Lat clones 15.4 and 8.4 in contrast to what we observed in the U1 cell line, indicating that the combinations can present a certain specificity and underlining the importance of testing the different combinations.

Our results showed that the non-tumor-promoting phorbol ester prostratin synergistically reactivated HIV-1 production with clinically used HDACIs (VPA, SAHA and NaBut) in monocytic and lymphocytic postintegration latency model systems, the two major cellular reservoirs in the natural host. Importantly, we demonstrated using human uninfected CD8^+^-depleted PBMCs that prostratin did not increase the cytotoxicity of these HDACIs, except for the MS-275 which exhibited a slight increased cellular toxicity when combined with prostratin (Figure S2, compare panel A and B; Text S2).

### Cotreatment with prostratin+HDACI induces HIV-1 expression in a higher proportion of J-Lat T cells than the drugs alone

**Figure 3:**
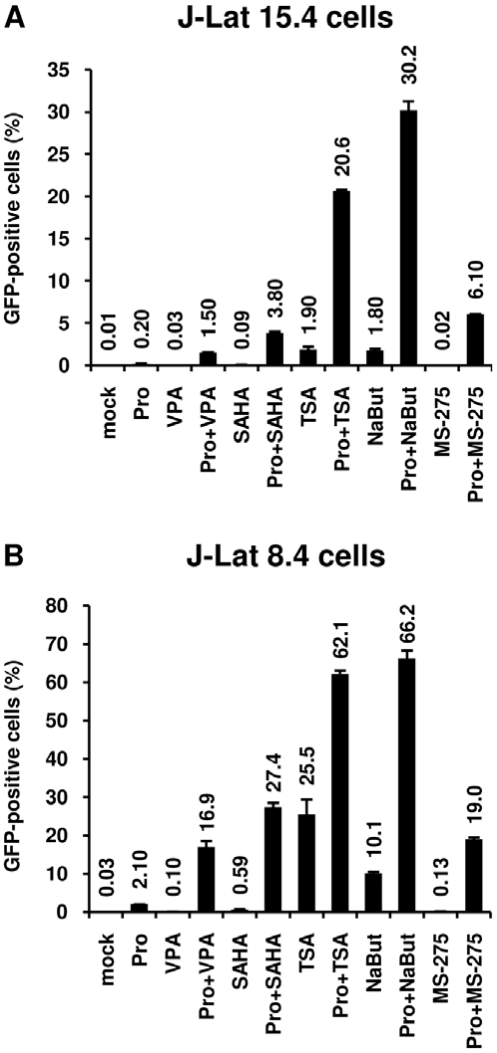
Prostratin+HDACI cotreatment induces HIV-1 expression in a higher proportion of cells than the drugs alone. J-Lat 15.4 (panel A) or 8.4 (panel B) cells were mock-treated or treated with prostratin (5 µM), alone or in combination with different HDACIs [VPA (2.5 mM), SAHA (2.5 µM), TSA (500 nM), NaBut (5 mM) or MS-275 (5 µM)]. At 24 h posttreatment, cells were analyzed by FACS for GFP expression and results (percentage of GFP-positive cells) are represented as histograms. Each value is the mean±SE from two separate experiments performed in duplicate.

In order to assess if the synergistic effect observed with a prostratin+HDACI cotreatment on viral p24 antigen production was due to an enhanced HIV-1 expression from those cells whose transcription was already reactivated by the drugs used alone or to the recruitment of unresponsive cells into the responding population, we used J-Lat T cells. As these cells harbor a full-length latent HIV-1 provirus containing GFP in place of *nef*, transcriptional activation of the latent provirus can be readily detected in individual cells by flow cytometry. As shown in Figure 3, in the absence of stimulation, the J-Lat 15.4 and 8.4 cells expressed no GFP, indicating a blockage of viral transcription. Stimulation of the J-Lat 15.4 cells with prostratin, VPA, SAHA, TSA, NaBut and MS-275 induced GFP expression in a small proportion of cells (0.20%, 0.03%, 0.09%, 1.90%, 1.80% and 0.02% of cells, respectively) (Figure 3A). Importantly, the proportion of J-Lat 15.4 cells displaying GFP epifluorescence was strongly and synergistically increased by the prostratin+HDACI cotreatments (Figure 3A). Similar results were obtained with the J-Lat clone 8.4 (Figure 3B and Figure S3).

These results indicated that, in the Jurkat CD4^+^ T-cell-based J-Lat clones, the prostratin+HDACI combinatorial treatment caused the synergistic recruitment of unresponsive J-Lat cells into the expressing cell population.

### Synergistic activation by prostratin and VPA of LTR activity from most of HIV-1 subtypes of the HIV-1 major (M) group

The HIV-1 group M isolates, which are responsible for more than 99% of all infections, have diversified during their worldwide spread. These isolates have been grouped according to their genomic sequences and are divided into 9 distinct subtypes termed A, B, C, D, F, G, H, J and K that can be further divided into sub-subtypes [53]. Moreover, when an identical recombinant virus is identified in at least three epidemiologically unlinked people and is characterized by full-length genome sequencing, it can be designated as a circulating recombinant form (CRF) [54]. More than 20 CRFs have been reported such as CRF01_AE (originally defined as subtype E) or CRF02_AG, recombinations of subtype A and E or A and G, respectively [53], [54].

**Figure 4:**
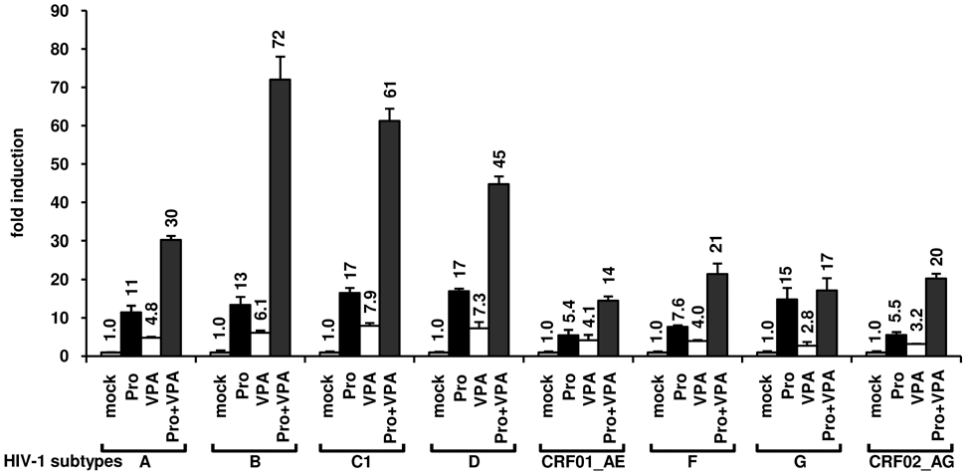
Effect of prostratin+VPA on transcriptional activity of LTRs from several HIV-1 major (M) group subtypes. Jurkat cells were transiently transfected with LTR-luciferase reporter constructs (LTRs from subtypes A, B, C1, D, CRF01_AE, F, G, CRF02_AG). At 20 h posttransfection, transfected cells were mock-treated or treated with prostratin (5 µM), VPA (2.5 mM), or prostratin+VPA. At 42 h posttransfection, cells were lysed and assayed for luciferase activity. Luciferase activities derived from the HIV-1 LTRs were normalized with respect to protein concentration using the Detergent-Compatible Protein Assay. Results are presented as histograms indicating inductions by the compounds (in fold) with respect to the basal activity of each pLTR-luc construct, which was assigned a value of 1. Each value is the mean±SE from two separate experiments performed in triplicate.

Functional distinctions in LTR architecture among HIV-1 subtypes have been identified, thus raising the possibility that regulatory divergence among the subtypes of HIV-1 has occurred. To examine the impact of these differences among the LTR sequences on the prostratin+VPA synergism, we performed transient transfections of Jurkat cells with reporter luciferase constructs containing LTRs from subtypes A, B, C1, D, F, G, and from CRF01_AE and CRF02_AG. Transfected LTRs were assayed for their responsiveness to prostratin alone, to VPA alone, or to both agents in combination. Results presented in Figure 4 show the induction for each subtype (obtained by dividing the induced luciferase activities of subtype X by the basal activity of this same subtype X) in order to eliminate subtype-specific differences in basal activity of the LTRs. LTR activity of each subtype tested was induced by prostratin alone from 5.4- to 17-fold and by VPA alone from 2.8- to 7.9-fold depending on the subtype (Figure 4). Importantly, prostratin and VPA together synergized (inductions from 14- to 72-fold) to activate all the subtype LTRs, except the subtype G LTR.

In conclusion, we showed that the combination prostratin+VPA synergistically activated transcription from LTRs belonging to several group M subtypes, including subtypes A, B, C, which represent the most prevalent HIV-1 genetic forms (subtype C accounting for almost 50% of all HIV-1 infections worldwide) [54]. These results were reminiscent of our previous studies using TNFα and TSA [3].

### VPA potentiates prostratin-induced nuclear NF-κB DNA-binding activity and cytoplasmic IκBα degradation

**Figure 5:**
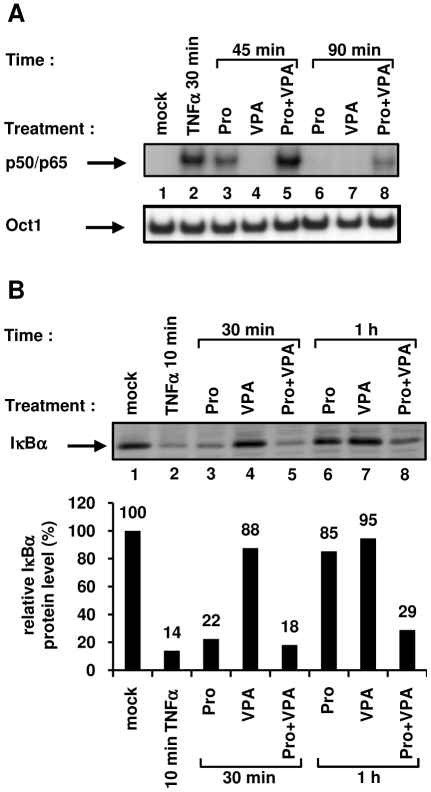
Effect of VPA on prostratin-mediated NF-κB signaling pathway activation. Nuclear (A) or cytoplasmic (B) extracts were prepared from Jurkat cells mock-treated or treated with TNFα (10 ng/ml), prostratin (5 µM), VPA (2.5 mM) or with prostratin+VPA for different time periods. A. An oligonucleotide corresponding to the HIV-1 LTR NF-κB sites was used as probe in EMSAs with the nuclear extracts. As control for equal loading, the bottom panel shows comparability of the various nuclear extracts assessed by EMSA with an Oct-1 consensus probe. An experiment representative of three independent experiments is shown. B. Cytoplasmic extracts were analyzed by Western blotting using an anti-IκBα antibody (top panel) and an anti-actin antibody (data not shown) as internal control. Levels of IκBα and actin were quantified by chemiluminescence analysis using ChemiDoc XRS System (Biorad) and results are expressed as IκBα amount/actin amount (bottom panel). The ratio obtained with the mock-treated Jurkat cells was arbitrarily assigned a value of 100%. An experiment representative of two independent experiments is shown.

In order to assess the molecular mechanisms responsible for the synergistic reactivation of HIV-1 production we observed following a prostratin+VPA cotreatment, we analyzed the effects of VPA on prostratin-induced NF-κB DNA-binding activity. To this end, we performed electrophoretic mobility shift assays (EMSAs) using an HIV-1 NF-κB probe and nuclear extracts from Jurkat T cells (Figure 5A). As expected, a rapid and strong NF-κB DNA-binding activity was observed in response to a 30 min treatment with TNFα (Figure 5A, lane 2) and VPA alone caused no induction of NF-κB binding activity even after a 90 min treatment (Figure 5A, lanes 4 and 7). Prostratin-induced NF-κB DNA-binding activity was weaker and had a slower kinetics than that induced by TNFα (Figure 5A, compare lane 3 with lane 2), in agreement with a previous report [28]. Supershift assays indicated that the observed retarded complex corresponded to the p50/p65 heterodimer (data not shown). When prostratin was combined with VPA, an induction of NF-κB binding activity stronger than that obtained with prostratin alone was observed at the two time points tested (Figure 5A, compare lane 5 with lane 3, and lane 8 with lane 6). Noteworthy, whereas prostratin-induced NF-κB DNA-binding activity faded away after a 90 min-treatment, VPA prolonged this activity (Figure 5A, compare lane 6 with lane 8).

NF-κB activation requires signal-coupled phosphorylation and degradation of the inhibitors of κB (IκBs, including IκBα), which bind and sequester NF-κB dimers in an inactive form in the cytoplasm of resting cells [55]. In order to determine whether the increased nuclear NF-κB DNA-binding activity observed in response to prostratin+VPA versus prostratin treatment resulted from an increased degradation of cytoplasmic IκBα, we monitored the presence of IκBα as a function of time in the cytoplasm after treatment with TNFα, prostratin, VPA or prostratin+VPA. TNFα induced a strong IκBα degradation after a 10 min treatment (Figure 5B, lane 2), whereas VPA alone had little effect on IκBα cytoplasmic levels (Figure 5B, lanes 4 and 7). A 30 min-treatment with prostratin induced IκBα degradation to a similar extent than a 10-min treatment with TNFα (Figure 5B, compare lane 3 with lane 2), thus confirming that prostratin activate the NF-κB pathway with a delayed kinetics compared to TNFα. Remarkably, whereas prostratin-induced IκBα degradation was followed by its reappearance after a 1 h treatment (Figure 5B, lane 6), complete reappearance of cytoplasmic IκBα at this time point was not detected after the combined treatment prostratin+VPA (Figure 5B, lane 8).

Our results demonstrated that VPA prolonged and increased prostratin-induced NF-κB DNA-binding activity and that this correlated with a prolonged IκBα degradation. This could explain, at least in part, the transcriptional synergism we observed between prostratin and VPA on the HIV-1 promoter.

### The combined treatment prostratin+VPA causes a more rapid and pronounced nucleosomal remodeling than the compounds alone

**Figure 6:**
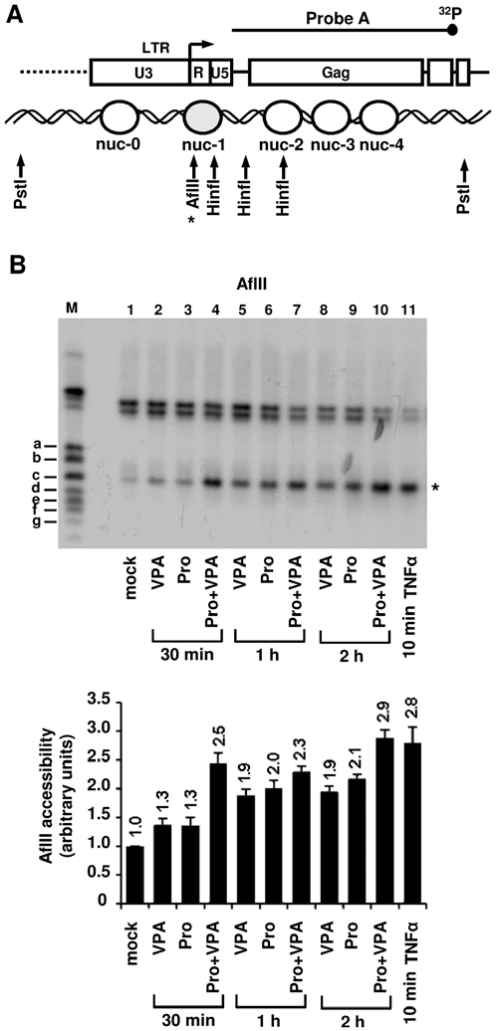
Remodeling of nuc-1 after prostratin+VPA versus VPA treatment. (A) Diagram indicating the positions of nucleosomes in the 5′ portion of the HIV-1 genome, the HinfI and AflII (*) cutting sites and the probe used in indirect end-labeling. An asterisk, representing the AflII cutting site, is located next to the band on the gel to permit its identification. (B) Nuclei were prepared from U1 cells mock-treated or treated with TNFα (10 ng/ml) (10 min), prostratin (5 µM), VPA (2.5 mM) and prostratin+VPA for 30 min, 1 h or 2 h and digested with AflII. After DNA purification and *in vitro* restriction with PstI, DNA samples were analyzed by indirect end-labeling using probe A (top panel) [93]. Size markers (a, b, c, d, e, f, g) have been previously described [93]. Quantification of the PstI-AflII bands was performed by radioimaging analysis using an InstantImager (Packard) (bottom panel) and results are presented as histograms indicating the band intensities relative to the intensity observed with mock-treated U1 cells, which was arbitrarily assigned a value of 1. Each value is the mean±SE of three separate experiments performed in duplicate.

We have previously reported that a single nucleosome (nuc-1) located immediately downstream of the HIV-1 transcription start site is remodeled during transcriptional activation of the HIV-1 promoter and that this remodeling is necessary for viral transcription [4], [5], [56]. To examine the effects of prostratin+VPA on nuc-1 remodeling, we determined the ability of the restriction endonuclease AflII, cutting within nuc-1 DNA, to cut genomic U1 DNA in the context of intact nuclei. The enzyme was incubated with purified nuclei from U1 cells mock-treated or treated as indicated in Figure 6B. DNA was purified and digested *in vitro* with PstI and analyzed by Southern blotting using indirect end-labeling.

At each time point tested, the combined treatment prostratin+VPA provoked an increase in nuc-1 DNA accessibility more pronounced than the increases observed after treatment with each drug individually (Figure 6B, compare lane 4 with lanes 2 and 3, lane 7 with lanes 5 and 6, and lane 10 with lanes 8 and 9). Of note, the 2 h combined treatment resulted in nuc-1 remodeling to the same extent as the control TNFα treatment at 10 min (Figure 6B, compare lane 10 with lane 11). Similar results were obtained with HinfI, another restriction endonuclease cutting within nuc-1 DNA and at two additional sites, thereby generating three bands (named x, y and z; Figure S4A and S4B) on Southern blots under incomplete digestion. The accessibility of these two additional sites (Figure S4B, bands y and z) remained unaffected by the different treatments.

In order to further examine, at the chromatin level, the molecular mechanisms involved in the prostratin+VPA synergism, we analyzed levels of histone H4 acetylation (an activation mark) in the nuc-1 region. To this end, we performed chromatin immunoprecipitation (ChIP) assays using U1 cells mock-treated or treated for different time periods with prostratin and VPA alone or in combination (Figure S5 and Text S3). VPA induced nuc-1 histone H4 acetylation, a result in agreement with previous reports [14], [57]. Prostratin slightly and gradually induced histone H4 acetylation starting at the 20 min treatment up to the 2 h treatment. This is in agreement with a previous study demonstrating that during HIV-1 transcriptional activation by the phorbol ester PMA, histone acetyltransferases are recruited to the viral promoter via NF-κB [58]. Importantly, our results indicated that, at each time point tested, the combined treatment prostratin+VPA did not induce a level of acetylated histone H4 higher than the level observed after treatments with VPA alone (Figure S5), suggesting that the more pronounced nuc-1 remodeling we observed by indirect end-labeling following combined versus individual treatment was not due to a cooperative induction of histone H4 acetylation.

These results demonstrated that the combined treatment prostratin+VPA resulted in a more rapid and pronounced nuc-1 remodeling than the treatments with the compounds alone.

### The combined treatment prostratin+VPA synergistically induces transcription from the viral LTR

**Figure 7:**
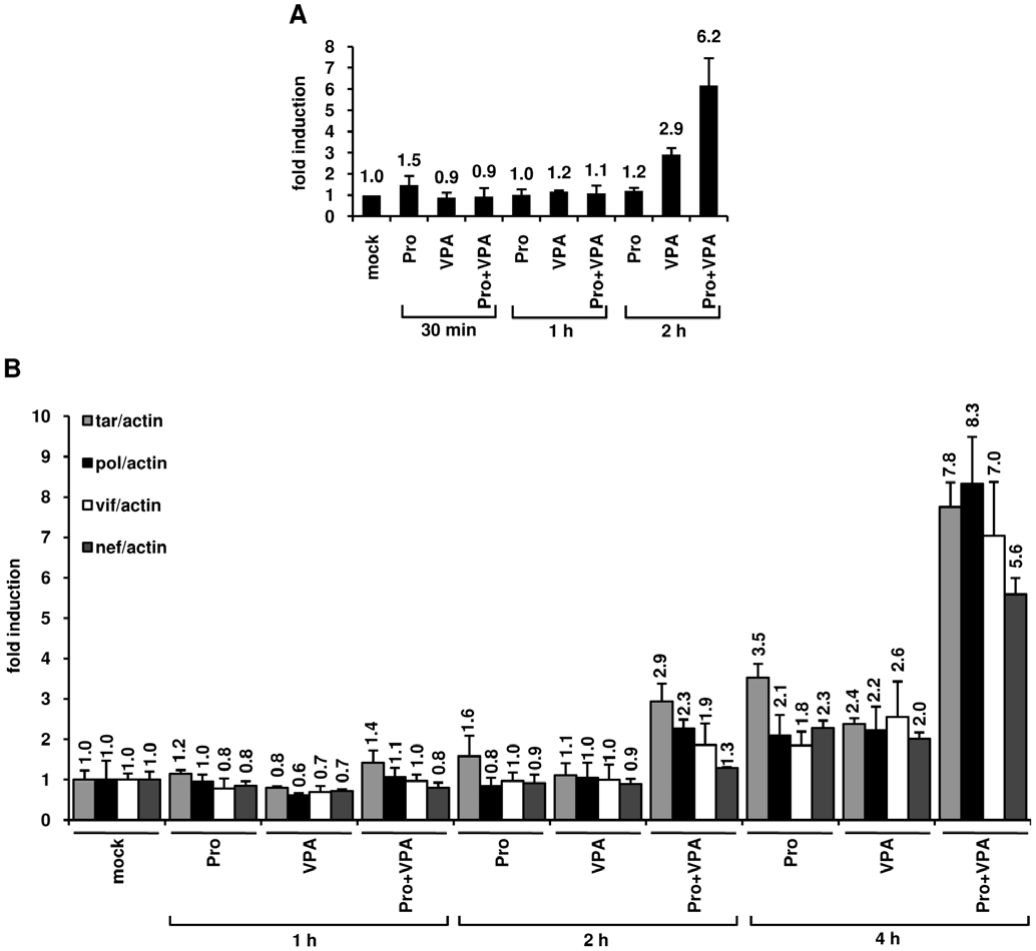
Effects of prostratin+VPA on HIV-1 transcription. (A) RNAPII occupancy of the HIV-1 promoter region was assessed by ChIP experiments. U1 cells were mock-treated or treated with prostratin (5 µM), VPA (2.5 mM) and prostratin+VPA for 30 min, 1 h or 2 h. The proteins were cross-linked with formaldehyde for 10 min and DNA sheared. The cross-linked protein/DNA complexes were immunoprecipitated with an anti-RNAPII antibody. The protein/DNA cross-links were reversed and the purified DNA was amplified and quantified by real-time PCR using primers amplifying the region overlapping the LTR U5 region and the primer binding site. Values were normalized to those obtained with the 18S rRNA primers and expressed as fold inductions relative to the value measured in mock-treated U1 cells, which was arbitrarily set at a value of 1. Each value is the mean±SE from three separate experiments performed in triplicate. (B) Measurement of initiated and elongated HIV-1 transcripts following drug treatments. Total RNA was extracted from U1 cells mock-treated or treated with prostratin (5 µM), VPA (2.5 mM) and prostratin+VPA for 1 h, 2 h or 4 h. Initiated (primers tar) or elongated (primers pol, vif and nef) transcripts were quantified by quantitative real-time RT-PCR. Values were normalized to those obtained with the β-actin primers and expressed for each primer pair as fold inductions relative to the values measured in mock-treated U1 cells, which were arbitrarily set at a value of 1. The results shown are the average of eight independent quantitative real-time PCRs from four separate reverse transcriptions from two independent cell culture treatments. The error bars show the standard errors. An experiment representative of two independent experiments is shown.

In order to test the effect of prostratin+VPA treatment on transcriptional activation of the HIV-1 promoter, we first monitored the RNA polymerase II (RNAPII) abundance in the 5'LTR region. To this end, we performed ChIP assays in U1 cells after treatment with prostratin alone, VPA alone or prostratin+VPA (Figure 7A). We used a primer pair overlapping the 3′ extremity of the LTR U5 region and the 5′ extremity of the leader region allowing to specifically determine RNAPII occupancy at the 5'LTR. After a 2 h combined treatment, we observed a cooperative effect of prostratin and VPA on RNAPII recruitment to the HIV-1 promoter and thus likely on transcription (Figure 7A).

To explore the functional relevance of this cooperative recruitment, we quantified initiated and elongated HIV-1 transcripts in U1 cells mock-treated or treated with prostratin, VPA or combination of both for different time periods (1 h, 2 h, 4 h). Initiated versus elongated HIV-1 transcripts were measured by quantitative real-time RT-PCR with primer sets targeting four different proviral regions: 1) TAR in the 5′ 60 bp; 2) *pol* gene located roughly 2.9 kb downstream of the 5'LTR; 3) *vif* gene located roughly 4.4 kb downstream of the 5'LTR; 4) *nef* gene, located roughly 8.3 kb downstream of the HIV-1 LTR. Theses primer sets allow analysis of transcriptional initiation and elongation at progressive positions throughout the HIV-1 provirus. As shown in Figure 7B, after a 4 h treatment with prostratin and VPA alone, we observed increased relative amounts of both initiated and elongated transcripts. Importantly, at the same time point, the prostratin+VPA combined treatment caused a synergistic accumulation of initiated and elongated transcripts (Figure 7B). Indeed, the prostratin+VPA cotreatment induced a 7.8-fold increase in initiated transcripts and 8.3-fold (pol probe), 7.0-fold (vif probe) and 5.6-fold (nef probe) increases in elongated transcripts compared to the mock-treated cells.

Altogether, our results measuring the RNAPII recruitment to the HIV-1 promoter and the amount of transcripts demonstrated that the prostratin+VPA synergism took place, at least in part, at the transcriptional initiation and elongation levels and demonstrated a temporal correlation between the degree of nuc-1 remodeling and the level of HIV-1 transcription.

### Prostratin and HDACIs induce HIV-1 recovery in CD8^+^-depleted PBMCs and in HLA DR^−^ CD4^+^ T cells from HIV-1 positive individuals with undetectable viral load

Our results suggested that a combination of prostratin+HDACI (such as VPA, SAHA or NaBut) could have a higher potential than the compounds alone in reactivating HIV-1 expression from cells isolated from HIV-1-infected individuals with undetectable viral load (<50 copies HIV-1 RNA/ml of plasma). To evaluate this hypothesis, we purified PBMCs from blood of 52 selected patients (Materials and Methods) and depleted them for CD8^+^ T cells, CD8^+^ T cells having an antiviral activity [59]. Importantly, these purified cells were cultured both in the absence of IL-2 and in the absence of allogenic stimulation (i.e. coculture with PBMCs from an uninfected individual) to avoid extensive nonspecific T-cell activation and proliferation, and thus resulting in no amplification of the genomic viral RNA level. After a one-day culture, cells were mock-treated or treated with anti-CD2+anti-CD28 antibodies (as a positive control), prostratin, HDACIs (VPA or SAHA) or with combinations of prostratin+HDACIs. Six days after treatment, we measured HIV-1 genomic RNA concentrations in culture supernatants.

For 10 out of 52 CD8^+^-depleted PBMC cultures, we detected viral RNA in the supernatant even in the absence of any treatment. Therefore, these data were removed from our study. Characteristics of the 42 remaining patients (named X1 to X42) are presented in Table 1. This table also indicates, for each PBMC culture, the combination of prostratin+HDACI tested (prostratin+VPA and/or prostratin+SAHA) and the levels of rescue obtained after anti-CD2+anti-CD28 treatment. These levels of rescue showed that the 17 CD8^+^-depleted PBMC cultures, presenting no viral outgrowth following treatment with prostratin and HDACI(s) individually or in combination, exhibited no or very weak levels of genomic viral RNA after anti-CD2+anti-CD28 costimulation compared to the levels observed with the PBMC cultures presenting viral reactivation after drug treatments (Table 1, compare patients X1 to X25 with X26 to X42). For 25 of the 42 PBMC cultures, we detected HIV-1 reactivation after prostratin, VPA, SAHA and/or prostratin+HDACI exposure (Tables 1 and 2, patients X1 to X25). We did not observe any significant correlation between age, CD4 counts or length of undetectable viremia, and the potency of our compounds to reactivate HIV-1 from latency. In 17 of these 25 cultures, prostratin alone reactivated viral expression, thereby confirming its great potential as an HIV-1 replication inducer (Table 2, X1, X2, X4 to X6, X9, X11, X14, X16, X18 to X25). VPA and SAHA alone reactivated viral expression in only 3 cell cultures, (Table 2, X4, X11, X14 and X4, X23, X24, respectively). In 8 cultures (Table 2, X3, X7, X8, X10, X12, X13, X15, X17), no drug used alone could reactivate viral expression.

**Table 1:** Characteristics of HIV-1-infected individuals named X1 to X42.

Patients	Age (years)	CD4 count at the time of the study (cells/µl)	Antiviral therapy at the time of the study^a^	Duration of therapy (years)	Duration with undetectable plasma HIV-1 RNA level (<50 copies/ml of plasma) (years)	Reactivation^b^	HIV-1 RNA level after treatment with anti-CD2+anti-CD28 antibodies^c^ (copies/ml)	Combination of prostratin+HDACI tested
X1	49	441	CBV, NVP	5	5	+	4070	Pro+VPA
X2	52	1000	ATV, EFV, RTV, TDF	8	3	+	42000	Pro+VPA
X3	56	596	CBV, DDI, fAPV	8	4	+	3920	Pro+VPA/Pro+SAHA
X4	45	1064	3TC, fAPV, RTV, TDF	13	2	+	39800	Pro+VPA/Pro+SAHA
X5	29	479	3TC, DDI, EFV	2	2	+	1253	Pro+VPA
X6	67	717	CBV, EFV	12	4	+	52300	Pro+VPA
X7	52	724	TZV	10	8	+	98000	Pro+VPA/Pro+SAHA
X8	45	542	3TC, EFV, fAPV, RTV, TDF	10	3	+	232	Pro+VPA/Pro+SAHA
X9	35	962	RTV, fAPV, CBV	12	1	+	467	Pro+VPA/Pro+SAHA
X10	39	558	TZV	13	8	+	241	Pro+VPA/Pro+SAHA
X11	50	786	TDF, FTC, EFV	2	2	+	6665	Pro+VPA/Pro+SAHA
X12	42	658	TZV	5	4	+	121	Pro+VPA/Pro+SAHA
X13	38	1239	NVP, TDF, 3TC	9	8	+	219	Pro+VPA/Pro+SAHA
X14	66	1073	TDF, 3TC, RTV, ATV	10	1	+	5053	Pro+VPA/Pro+SAHA
X15	50	1748	CBV, NVP	11	10	+	248	Pro+SAHA
X16	40	722	RTV, fAPV, TDF, FTC	2	2	+	72	Pro+SAHA
X17	42	614	3TC, TDF, NVP	4	3	+	105	Pro+SAHA
X18	66	842	3TC, ABC, EFV	4	1	+	172	Pro+VPA
X19	57	587	CBV, EFV, LPV/r	8	3	+	3154	Pro+VPA
X20	62	437	D4T, KVX, NVP	13	7	+	11200	Pro+VPA/Pro+SAHA
X21	52	934	3TC, ABC, fAPV, RTV	8	4	+	6478	Pro+VPA
X22	43	680	NVP, fAPV, KVX, RTV	12	6	+	15394	Pro+VPA/Pro+SAHA
X23	30	536	RTV, fAPV, FTC, TDF	2	2	+	611	Pro+VPA/Pro+SAHA
X24	66	1183	ATV, 3TC, TDF	13	1	+	3121	Pro+SAHA
X25	34	721	3TC, TDF, NVP	11	10	+	13465	Pro+SAHA
X26	29	617	3TC, ABC, LPV/r	1	1	-	0	Pro+VPA
X27	54	689	3TC, TDF	9	2	-	0	Pro+VPA
X28	36	774	CBV, NVP	4	4	-	0	Pro+VPA
X29	62	611	TZV	8	2	-	99	Pro+VPA
X30	61	502	LPV/r, NVP	6	2	-	360	Pro+VPA
X31	55	959	TZV	5	5	-	0	Pro+VPA/Pro+SAHA
X32	27	501	3TC, ABC, EFV	3	3	-	0	Pro+VPA
X33	61	508	EFV, KVX, TDF	9	6	-	102	Pro+VPA
X34	46	515	TZV	9	9	-	0	Pro+VPA
X35	58	490	CBV, NVP	8	3	-	142	Pro+VPA/Pro+SAHA
X36	51	682	IDV, NVP, RTV	10	5	-	463	Pro+VPA/Pro+SAHA
X37	34	599	CBV, fAPV, RTV	6	6	-	299	Pro+VPA
X38	44	1048	AZT, DDI	11	10	-	0	Pro+VPA/Pro+SAHA
X39	38	857	FTC, TDF, EFV	5	4	-	0	Pro+SAHA
X40	40	798	EFV, KVX	4	2	-	0	Pro+SAHA
X41	60	697	RTV, ATV, CBV	11	5	-	1241	Pro+SAHA
X42	43	643	NVP, 3TC, TDF	5	5	-	0	Pro+SAHA
Mean	47.7	746		7.6	4.2			
Range	27–67	437–1748		1–13	1–10			

a Nucleoside analogs: Abacavir (ABC), Didanosine (DDI), Stavudine (D4T), Lamivudine (3TC), Zidovudine/Lamivudine (CBV), Zidovudine/Lamivudine/Abacavir (TZV), Tenofovir (TDF), Abacavir/Lamivudine (KVX), Emtricitabine (FTC), Zidovudine (AZT). Nonnucleoside reverse transcriptase inhibitors: Nevirapine (NVP), Efavirenz (EFV). Protease inhibitors: Indinavir (IDV), Ritonavir (RTV), Lopinavir/Ritonavir (LPV/r), Atazanavir (ATV), Fosamprenavir (fAPV).

b Reactivation of HIV-1 expression (+).

c level of HIV-1 expression were determined by measuring HIV-1 RNA levels as described in Materials and Methods.

**Table 2:** Reactivation profiles observed in CD8^+^-depleted PBMC cultures from patients X1 to X25.

Treatments	Pro	VPA	Pro+VPA	SAHA	Pro+SAHA
Patients					
X1	+	−	+*	/	/
X2	+	−	+*	/	/
X3	−	−	−	−	+*
X4	+	+	+*	+	+*
X5	+	−	+*	/	/
X6	+	−	+*	/	/
X7	−	−	−	−	+*
X8	−	−	−	−	+*
X9	+	−	+*	−	+*
X10	−	−	+*	−	+*
X11	+	+	+*	−	+*
X12	−	−	+*	−	+*
X13	−	−	+*	−	+*
X14	+	+	+*	−	+*
X15	−	/	/	−	+*
X16	+	/	/	−	+*
X17	−	/	/	−	+*
X18	+	−	+	/	/
X19	+	−	+	/	/
X20	+	−	+	−	+
X21	+	−	+	/	/
X22	+	−	+	−	+
X23	+	−	+	+	+
X24	+	/	/	+	+
X25	+	/	/	−	+
Percentage of reactivation	68%	15%	85%	17%	100%

Cultures were mock-treated or treated with prostratin (5 µM), VPA (2.5 mM) or SAHA (2.5 µM) alone or in combination. Reactivation of HIV-1 expression (+), synergistic viral reactivation (+*) or no viral outgrowth (−) were determined by measuring HIV-1 RNA levels as described in Materials and Methods. For some patients, insufficient number of CD8^+^-depleted PBMCs was available for testing the considered conditions (/).

**Figure 8:**
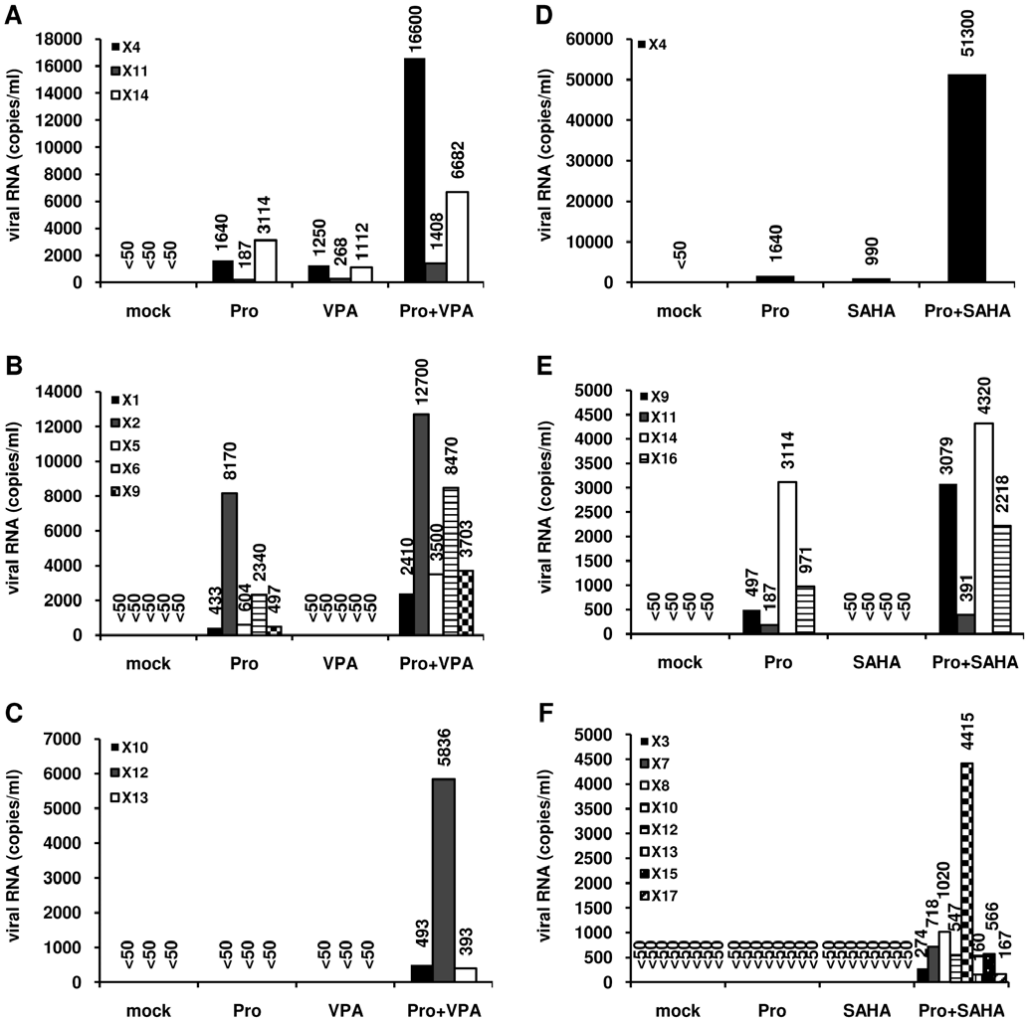
Prostratin+HDACIs synergistically induce HIV-1 recovery in CD8^+^-depleted PBMCs from HAART-treated patients with undetectable viral load. CD8^+^-depleted PBMCs from patients X1 to X17 were mock-treated or treated with prostratin (5 µM), VPA (2.5 mM) (panels A, B and C), SAHA (2.5 µM) (panels D, E and F) alone or in combination. Six days after treatment, HIV-1 genomic RNA concentrations were measured in culture supernatants as described in Materials and Methods. When a single patient PBMCs culture presented distinct profiles depending on the treatment (prostratin+VPA or prostratin+SAHA), this culture was shown in several categories.

In 17 out of the 25 reactivated cultures, HIV-1 replication was synergistically induced by a combination prostratin+HDACI (Table 2, patients X1 to X17). These 17 CD8^+^-depleted PBMC cultures can be subdivided in three categories for each combination prostratin+HDACI (Figure 8A, 8B, 8C for prostratin+VPA and Figure 8D, 8E, 8F for prostratin+SAHA). The first category concerned 3 cultures for the combination prostratin+VPA (Figure 8A, X4, X11, X14) and one culture for the combination prostratin+SAHA (Figure 8D, X4). After treatment with prostratin, VPA or SAHA alone, we detected HIV-1 genomic RNA in the supernatants. After combined treatments with prostratin+VPA or prostratin+SAHA, we measured a synergistic increase in viral RNA copy number. The second category concerned CD8^+^-depleted PBMC cultures in which we detected HIV-1 reactivation after treatment with prostratin, prostratin+VPA (Figure 8B, X1, X2, X5, X6, X9) or prostratin+SAHA (Figure 8E, X9, X11, X14, X16) but not after treatment with VPA or SAHA alone. In the third category, we grouped the results obtained with CD8^+^-depleted PBMC cultures in which we detect viral RNA only after a combined treatment with prostratin+VPA (Figure 8C, X10, X12, X13) or prostratin+SAHA (Figure 8F, X3, X7, X8, X10, X12, X13, X15, X17). In cultures from patients X18 to X25, no synergistic effect of the drug combinations was observed (Table 2).

For the CD8^+^-depleted PBMC cultures from patients X26 to X42, we did not detect any viral outgrowth following treatment with prostratin and HDACIs individually or in combination (Table 1). However, we cannot exclude that, in some of these cultures, reactivation levels were under the detection limit of the Roche Amplicor assay.

**Figure 9:**
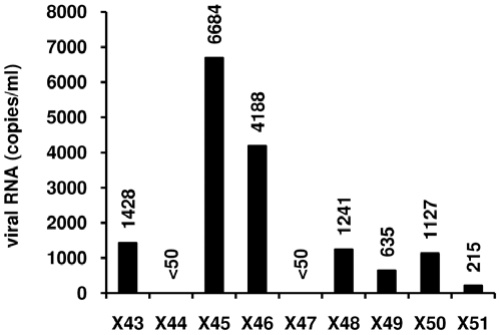
Effects of prostratin+SAHA treatment on HIV-1 recovery in HLA DR^−^ CD4^+^ T cells. HLA DR^−^ CD4^+^ T cells from HAART-treated patients with undetectable viral load (X43 to X51) were mock-treated or treated with the combination prostratin (5 µM)+SAHA (2.5 µM). Six days after treatment, HIV-1 genomic RNA concentrations were measured in culture supernatants as described in Materials and Methods.

Among the cell types present in CD8^+^-depleted PBMCs, latently-infected resting memory CD4^+^ T cells that harbor integrated replication-competent viral DNA represent the primary long-lived source of HAART-persistent HIV-1 [13], [25], [60]. These resting cells carrying a non-productive HIV-1 infection, derived from infected CD4^+^ lymphoblasts that have reverted back to a resting memory state and show a specific pattern of surface markers (including CD4^+^and HLA DR^−^) [13]. To evaluate in HLA DR^−^ CD4^+^ T cells the results we obtained in CD8^+^-depleted PBMCs, we purified HLA DR^−^ CD4^+^ T cells from blood samples obtained from 9 HAART-treated patients selected as described in Materials and Methods. Characteristics of patients named X43 to X51 are presented in Table 3. Because of limited amounts of HLA DR^−^ CD4^+^ T cells, we tested the reactivation effect of the combined treatment prostratin+SAHA, shown here above to reactivate HIV-1 expression with higher efficiency than the combination prostratin+VPA in CD8^+^-depleted PBMC cultures. We demonstrated that HIV-1 replication was reactivated in 7 HLA DR^−^ T-cell cultures out of 9 tested, with RNA viral loads ranging from 215 to 6684 copies/ml depending on the patient culture (Figure 9).

**Table 3:** Characteristics of HIV-1-infected individuals named X43 to X51.

Patients	Age (years)	CD4 count at the time of the study (cells/µl)	Antiviral therapy at the time of the study^a^	Duration of therapy (years)	Duration with undetectable plasma HIV-1 RNA level (<50 copies/ml of plasma) (years)
X43	47	325	TVD, RTV, ATV	9	2
X44	54	307	TVD, EFV	14	5
X45	49	1298	LPV/r, EFV, TVD	18	7
X46	37	461	TVD, EFV	8	7
X47	49	759	TVD, SQV, RTV	8	6
X48	41	707	EFV, LPV/r, FTC	13	7
X49	35	463	KVX, NVP	8	8
X50	35	841	LPV/r, TVD	2	2
X51	60	608	CBV, NVP	16	9
Mean	45.2	641		10.7	5.9
Range	35–60	307–1298		2–18	2–9

a Nucleoside analogs: Tenofovir/Emtricitabine (TVD), Emtricitabine (FTC), Zidovudine/Lamivudine (CBV), Abacavir/Lamivudine (KVX). Nonnucleoside reverse transcriptase inhibitors: Nevirapine (NVP), Efavirenz (EFV). Protease inhibitors: Ritonavir (RTV), Lopinavir/Ritonavir (LPV/r), Atazanavir (ATV), Saquinavir (SQV).

In our studies, we used 6×10^6^ CD8^+^-depleted PBMCs or 2×10^6^ HLA DR^−^ CD4^+^ T cells per well, therefore, there are probably very few latent cells in each well. It is possible that, in some cases, the differences in the HIV-1 recovery levels observed in the different cultures as well as the failure of the drugs alone or in combination to stimulate HIV-1 in some cultures may be due to partioning of the few infected cells. However, given the high number of CD8^+^-depleted PBMC cultures we tested (42) and the results obtained in several latently-infected cell lines, it seems unlikely that the results reported here would be due to random distributions of the infected cells between culture wells.

In conclusion, although the HIV-1 reactivation levels in response to the different treatments varied importantly from one patient to the other, the clinically used HDACIs (VPA or SAHA) alone or the non-tumor-promoting phorbol ester prostratin alone induced HIV-1 outgrowth in CD8^+^-depleted PBMC cultures in the absence of added IL-2 or of allogenic stimulation. Importantly, the combination prostratin+HDACI allowed HIV-1 reactivation in a higher number of cell cultures compared to the number of positive cultures observed with the activators individually, and to a higher extent than these activators alone. Moreover, the combined treatment prostratin+SAHA efficiently reactivated viral replication in resting CD4^+^ T cells, which constitute the most stable HIV-1 reservoir.

## Discussion

The HIV-1 latent reservoirs decrease only slowly in patients undergoing HAART. It is estimated that decades of treatment would be required to completely eliminate the latent virus. Ideal compounds for viral purging would induce viral replication in a wide variety of infected cell types without causing massive T-cell activation and proliferation to prevent the generation of new target cells for the neosynthetized virus, while maintaining the patient on HAART to prevent a spreading infection. In this report, we combined two different types of compounds that meet these criteria: the non-tumor-promoting NF-κB inducer prostratin and HDACIs already in use for other diseases and thus clinically promising for HIV-1 flushing strategies. We demonstrated a synergistic activation of HIV-1 production by combinations of prostratin+HDACI in the latently-infected monocytic cell line U1 and T-lymphoid J-Lat 8.4 and 15.4 cell lines. In these later cell lines, we showed that the synergistic effect was due, at least partially, to the recruitment of unresponsive cells into the expressing cell population. VPA increased prostratin-induced NF-κB activation and potentiated nuc-1 remodeling. The prostratin+VPA combined treatment caused a synergistic accumulation of initiated and elongated transcripts, as demonstrated by quantitative real-time RT-PCR. We performed viral reactivation assays in cell cultures prepared from HAART-treated HIV-1-infected individuals with undetectable viral load and showed that a combination prostratin+VPA or prostratin+SAHA induced HIV-1 recovery in 25 of the 42 CD8^+^-depleted PBMC cultures tested (with a synergistic recovery observed in 17 out of these 25). Moreover, the combination prostratin+SAHA reactivated viral replication in 7 of the 9 HLA DR^−^ CD4^+^ T cell cultures prepared from additional patients. This study suggests that this type of combinatory activation approach could be used as an inducible adjuvant therapy with efficient HAART to accelerate latent HIV-1 clearance.

Viral reactivation trials with activating agents such as IL-2 and OKT3 (a murine monoclonal anti-CD3 antibody), have shown little, if any, reduction in viral rebound after cessation of HAART [60] and their *in vivo* application has resulted in deleterious side effects [61]. In 2002, we proposed the use of HDACIs as an inductive adjuvant therapy with efficient HAART aimed at forcing HIV-1 gene expression in the latently-infected cells [3], [10]. Here, we demonstrated that the 11 inhibitors of class I and II HDACs (but not the two inhibitors of class III HDACs we tested) all induced HIV-1 production in U1 cells, independently of the structural group these HDACIs belonged to. This is in agreement with data from several groups reporting the recruitment of class I HDACs to the HIV-1 promoter [62], [63], [64], [65], [66].

Because VPA is in wide use to treat common chronic neurological and psychiatric disorders and often given simultaneously to HAART for long periods of time to HIV-1-infected individuals, many recent studies aimed at determining VPA potential to accelerate clearance of HIV-1. Whereas two works have suggested that the VPA could be a good candidate to deplete latent infection [14], [15], more recent studies did not confirm these results [18], [19], [20], [67] (and this paper). In agreement with this, our reactivation assays showed that VPA alone induced HIV-1 recovery in only 3 out of 33 CD8^+^-depleted PBMC cultures prepared from patients receiving HAART. However, our results indicated that VPA used in combination with prostratin was more efficient in reactivating HIV-1 replication than these compounds used individually. Indeed, in some cell cultures in which VPA alone did not cause any detectable HIV-1 reactivation, this HDACI potentiated prostratin-induced HIV-1 recovery. Moreover, in the 3 CD8^+^-depleted PBMC cultures in which VPA alone reactivated viral expression, we detected a synergistic recovery of HIV-1 RNA production after the combined treatment prostratin+VPA. Therefore, combined with other kinds of HIV-1 inducers, VPA could have an impact on the decay of latent reservoirs, despite its weak HDAC inhibitor activity. Its combined administration could be even more beneficial for long-term purging therapies than other FDA (Food and Drug Administration)-approved more potent HDAC inhibitors, such as SAHA that exhibits relative toxicities (diarrhea, fatigue, nausea, anorexia and dehydratation) [42], [49]. Such combination approaches could provide an important HIV-1 activation leading to a decline of HIV-1 reservoirs level sufficient to allow an efficient control of the infection by the host immune system, and might thus allow therapeutic interruptions (“treatment-free windows”).

Our FACS analyses of the J-Lat clones revealed that the proportion of J-Lat cells displaying GFP epifluorescence was synergistically increased by prostratin+HDACI cotreatments compared to treatments with the compounds alone. This implied that the synergistic effect observed following a prostratin+HDACI cotreatment on viral p24 antigen production was due, at least in part, to the recruitment of unresponsive cells into the responsive cell population. However, we cannot exclude that part of this synergistic effect also resulted from a higher level of expression from the already responding cell population. The cells containing HIV-1 promoters, that were reactivable neither by HDACI alone nor by prostratin alone but were reactivable by the combination, are likely further locked in an epigenetic silent state compared to the more easily reactivable promoters, which are responsive to a drug alone. Those J-Lat cells that remain unresponsive to the combined treatment are probably regulated by repressive epigenetic marks that cannot be reversed solely by inducing NF-κB and inhibiting HDAC activity.

Among the elements involved in HIV-1 reactivation, NF-κB activity is of paramount importance. Indeed, the two NF-κB-binding sites in the enhancer are critical for LTR promoter activity and important for optimal HIV-1 replication [11]. Here, we report that prostratin alone induced IκBα degradation and NF-κB DNA-binding activity, although the prostratin-induced activation of the NF-κB signaling pathway was weaker in intensity and had a delayed kinetics compared to that elicited by TNFα, in agreement with a previous study [28]. Importantly, we showed that VPA increased and prolonged prostratin-induced NF-κB DNA-binding activity. This is consistent with the fact that acetylation/deacetylation events regulate the NF-κB activating signaling pathway at multiple levels, including the direct acetylation of NF-κB [55]. Indeed, Lys221 acetylation enhances p65 DNA-binding activity and, together with acetylation at Lys218, impairs its assembly with IκBα, thereby impeding IκBα-dependent nuclear export of the NF-κB complex and enabling prolongation of the NF-κB response [55]. Moreover, the acetylated form of p50 binds target sequences with higher affinity than the unacetylated form does [55]. Noteworthy, the prolonged NF-κB DNA-binding activity that we observed here was correlated with a prolonged IκBα degradation. IκBα degradation is dependent upon its phosphorylation by the IκB kinase (IKK) complex and its subsequent ubiquitination. Thereby, VPA could prolong the prostratin-induced IκBα degradation by prolonging the IKK activity, as we have previously shown for the TNFα+TSA combined treatment [21].

The nucleosome nuc-1 is positioned immediately downstream of the HIV-1 transcription start and is remodeled upon transcriptional activation by Tat and the HDACIs TSA or Trapoxin in the absence of NF-κB stimulation [4], [5]. These observations suggest that nuc-1 plays a repressive role in HIV-1 transcription and that histone acetylation could be involved in overriding this effect. Accordingly, we reported here that the HDACI VPA induced nuc-1 histone H4 acetylation. Importantly, we showed that the combination prostratin+VPA did not induce a level of acetylated histone H4 higher than the level observed with VPA alone. Therefore, the cooperative nuc-1 remodeling we observed by indirect end-labeling following a prostratin+VPA cotreatment was not due to a cooperative induction of histone H4 acetylation. Chromatin structure can be altered both by histone posttranslational modifications (such as acetylation), which alter histone/DNA or histone/histone interactions, and by ATP-dependent chromatin remodeling complexes (such as SWI/SNF) [68]. It has been previously reported that these SWI/SNF complexes are recruited at gene promoters by transcription factors, including NF-κB [69], [70] and AP-1 [71], [72], or by acetylated histones [73]. Therefore, by inducing NF-κB and/or AP-1 binding to the HIV-1 5'LTR [22], [28], [32], prostratin could be involved in the remodeling of nuc-1 through the recruitment of SWI/SNF by these transcription factors. This hypothesis could explain the more pronounced nuc-1 remodeling we observed in the postintegration latency model cell line U1 after the prostratin+VPA cotreatment compared to the treatments by the drugs alone.

We demonstrated a temporal correlation between the degree of nuc-1 remodeling and the level of transcription in U1 cells by showing that the prostratin+HDACI-induced increase in chromatin remodeling at 2 h postinduction was correlated with an increased RNAPII recruitment to the viral LTR at the same time point and to an increased accumulation of both initiated and elongated transcripts at 4 h postinduction. Expression of full-length HIV-1 transcripts requires the action of the cellular kinase P-TEFb, composed of a catalytic subunit, cyclin-dependent kinase 9 (Cdk9) and a regulatory subunit, cyclinT1. The absence or inactivity of this protein, while generating short viral transcripts, fails to support effective viral replication. P-TEFb mediates transcriptional elongation of the bound polymerase complexes by phosphorylating the C-terminal domain (CTD) of RNAPII. Although P-TEFb is mainly recruited via the viral transactivator Tat, other mechanisms that can ensure P-TEFb recruitment and thereby transcriptional elongation have been described and can account for signal-induced transcription elongation in Tat-defective HIV-1 infected cells, such as the U1 cell line. Notably, Sp1, p65 and histone acetylation can contribute to P-TEFb recruitment to the HIV-1 promoter [74], [75], [76], [77], via Brd4, a positive regulatory component of P-TEFb, for p65 and histone acetylation [75], [76], [77]. Brd4 is a bromodomain-containing protein that recognizes acetylated histones [78] as well as acetylated p65 [75]. Altogether, these previous data could explain at the molecular level our quantitative real-time RT-PCR results showing that prostratin and VPA synergistically activated transcriptional elongation in U1 cells. The prostratin+VPA cotreatment could allow a more important recruitment of the Brd4/P-TEFb complex to the HIV-1 promoter than the drugs alone. In addition to activating the NF-kB signaling pathway, prostratin up-regulates cyclinT1 expression, leading to an increased association between cyclinT1 and cyclin-dependent kinase 9 (Cdk9) which results in a more efficient P-TEFb kinase activity [79].

Our reactivation assays suggested that when combined with prostratin, SAHA could be a more potent HIV-1 reactivating agent than VPA. Indeed, whereas a prostratin+VPA cotreatment reactivated viral expression in 17 out of the 33 CD8^+^-depleted PBMC cultures tested (11 presenting synergistic activation), a combined treatment with prostratin+SAHA reactivated viral expression in 18 out of 26 CD8^+^-depleted PBMC cultures (13 presenting synergistic activation) and in 7 out of the 9 HLA DR^−^ CD4^+^ T cell cultures we tested. Supporting our data, two very recent studies, published during the revision of the present paper, have reported that SAHA reactivates HIV-1 from latently-infected cells [80], [81]. SAHA is administered intravenously to patients with refractory hematologic and solid tumors and has been approved for use as a treatment of cutaneous T-cell lymphoma [42], [48], [49]. Because of its ability to promote HIV-1 expression, especially in combination with prostratin, SAHA provides additional promise for the decay of latent HIV-1 infection and deserves further investigations.

Of note, in 17 out of 42 CD8^+^-depleted PBMC cultures, we did not detect any viral outgrowth following treatment with prostratin and HDACIs individually or in combination and no or very weak viral outgrowth following an anti-CD2+anti-CD28 treatment compared to the levels observed with the PBMC cultures presenting viral reactivation after drug treatments. A possible explanation to the variegated response to prostratin+HDACI treatment observed in the CD8^+^-depleted PBMC cultures could result from an “on-off switching” of transcriptional competence. Local chromatin structure at the site of virus integration within the host genome modulates the level of epigenetic HIV-1 transcriptional repression as well as the ability of the virus to respond to reactivating stimuli [82]. Indeed, some of the integrated HIV-1 DNA may be under strong epigenetic repression (through DNA methylation and histone methylation and deacetylation) that cannot be efficiently reversed by the drugs we used in this study [7], [83]. However, we cannot rule out the possibility that the viruses present in the samples in which we could not detect any viral reactivation were defective. Therefore, locus-specific transcriptional repression might account, at least in part, for the heterogeneous HIV-1 reactivation profiles we observed after prostratin+HDACI cotreatment.

In conclusion, we report a proof-of-concept study for the therapeutic potential coadministration of two different kinds of HIV-1 activators (one acting on the NF-κB pathway and the other acting on the protein acetylation status) aimed at efficient decay of latent reservoirs in presence of HAART. However, we did not observe reactivation of viral replication in all the patient cell cultures tested, thereby emphasizing the importance to identify other combinations involving SAHA, prostratin, prostratin-like compounds with higher potency and/or other kinds HIV-1 inducers (such as inhibitors of histone- and DNA-methyltransferases). In this regard, a recent study has reported the practical synthesis in gram quantities and at low cost of prostratin, 12-deoxyphorbol-13-phenylacetate (DPP) and a variety of new 12-deoxyphorbol analogs, which might be potentially superior clinical candidates against latent HIV-1 [34].

## Materials and Methods

### Cell Lines and Cell Culture

The monocytic cell line U1 [84] and the T-lymphoid cell lines Jurkat [85], J-Lat 8.4 and J-Lat 15.4 [51] were obtained from the AIDS Research and Reference Reagent Program (National Institute of Allergy and Infectious Diseases [NIAID], National Institute of health [NIH]). All cell lines were grown in RPMI 1640 medium (Gibco-BRL) supplemented with 10% fetal bovine serum, 50 U/ml of penicillin, 50 µg/ml of streptomycin at 37°C in a humidified 95% air/5% CO_2_ atmosphere.

### Reagents

TNFα (catalog no. 300-01A) was purchased from Immunosource. SAHA (suberoylanilide hydroxamic acid) (catalog no. 270-288-M001) was obtained from Alexis Biochemichals. Prostratin (12-deoxyphorbol-13-acetate) (catalog no. PE 187-0001), HC-Toxin (Cyclo(D-prolyl-L-alanyl-D-alanyl-L-2-amino-9,10-epoxy-8-oxodecanoyl)) (catalog no. GR-320), SBHA (suberoyl bis-hydroxamic acid) (catalog no. GR-323), Scriptaid (6-(1,3-dioxo-1H,3H-benzo[de]isoquinolin-2-yl)-N-hydroxyhexanamide) (catalog no. GR-326) were provided by Tebu-Bio. NaBut (sodium butyrate) (catalog no. B5887), VPA (2-propylpentanoic acid sodium or valproic acid) (catalog no. P4543), Depudecin (D-threo-D-ido-undeco-1,6-dienitol,4,5:8,9-dianhydro-1,2,6,7,11-pentadeoxy) (catalog no. D5816), apicidin (cyclo[(2S)-2-amino-8-oxodecanoyl-1-methoxy-L-tryptophyl-L-isoleucyl-(2R)-2-piperidinecarbonyl]) (catalog no. A8851), TSA (trichostatin A) (catalog no. T8552) were purchased from Sigma-Aldrich. PMA (phorbol myristate acetate) (catalog no. 524400), Splitomycin (1,2-dihydro-3H-naphtho[2,1-b]pyran-3-one) (catalog no. 567750), MS-275 (N-(2-Aminophenyl)-4-[N-(pyridine-3-ylmethoxycarbonyl)aminomethyl] benzamide) (catalog no. 382147), Sirtinol (2-[(2-hydroxynaphthalen-1-ylmethylene)amino]-N-(1-phenethyl)benzamide) (catalog no. 566320), CBHA (m-carboxycinnamic acid bis-hydroxamide) (catalog no. 382148) were obtained from Calbiochem. All compounds, resuspended and stored as recommended by the manufacturer, were diluted immediately before use in cell culture medium.

### Virus production assays in U1 and J-Lat 8.4 or 15.4 cell lines

HIV-1 production was measured in the U1 and J-Lat 8.4 or 15.4 cell line culture supernatants by determining CA-p24 antigen concentration by an enzyme-linked immunosorbent assay (Innogenetics).

### Transient transfection and luciferase reporter assays

Jurkat cells were transfected with the pLTR (A, B, C1, D, CRF01_AE, F, G, CRF02_AG) –luciferase reporter plasmids (1000 ng) [86] using jetPEI™ (POLYplus) according to the manufacturer's protocol. Cells were mock-treated or treated with the different compounds as indicated, lysed and assayed for luciferase activity as previously described [3].

### EMSAs

Nuclear extracts were prepared and EMSAs with the HIV-1 NF-κB probe were performed as previously described [3]. As loading controls, the same nuclear extracts were tested for binding of Oct-1 (octamer-binding protein-1) to an Oct-1 consensus probe (5′-TGTCGA**ATGCAAAT**CACTAGAA-3′).

### Western blot analyses

Cytoplasmic extracts were prepared as previously described [87]. Western blots were performed [87] with a rabbit antibody against IκBα (1/1000 dilution; catalog no. 06-494; Upstate Biotechnology) or against actin (1/1000 dilution; catalog no.A2066; Sigma-Aldrich) and a peroxidase-conjugated goat anti-rabbit IgG (1/3000 dilution; catalog no. 7074; Cell Signaling).

### Indirect end-labeling technique

The indirect end-labeling experiments were performed as previously described [4].

### Study Subjects

We selected 52 HIV-1-infected individuals at the St-Pierre Hospital (Brussels, Belgium) and 9 additional patients at the Bicêtre Hospital (Bicêtre, France) on the basis of the following criteria: all volunteers were treated with HAART for at least 1 year, had an undetectable plasma HIV-1 RNA level (<50 copies/ml) for at least 1 year and had a level of CD4^+^ T lymphocytes higher than 300 cells/mm^3^ of blood. Characteristics (age, CD4^+^ T cell count, antiviral regimens, duration of therapy, duration with undetectable plasma HIV-1 RNA level) of patients from the St-Pierre Hospital and from the Bicêtre Hospital are presented in Table 1 and 3, respectively.

### Ethics Statement

Ethical approval was granted by the Human Subject Ethics Committees of the Saint-Pierre Hospital and of the Bicêtre Hospital. All individuals enrolled in the study provided written informed consent for donating blood.

### Isolation of CD8^+^-depleted PBMCs and of HLA DR^−^ CD4^+^ T lymphocytes

CD8^+^-depleted PBMCs were isolated from fresh whole blood (50 ml) of the HIV-1-infected individuals described above by adding RosetteSep human CD8 depletion mixture (StemCell Technologies) to whole blood samples before density centrifugation on a Ficoll-Hypaque gradient (Pharmacia). HLA DR^−^ CD4^+^ T lymphocytes were isolated as previously described [88]. The number of cells (CD8^+^-depleted PBMCs and HLA DR^−^ CD4^+^ T lymphocytes) obtained after isolation varied depending on the patients. In all cases, cells were washed with RPMI, resuspended at 2×10^6^ cells/ml of complete RPMI (RPMI, 10% fetal bovine serum, supplemented with 50 U/ml of penicillin, and 50 µg/ml of streptomycin). As a positive control of reactivation, CD8^+^-depleted PBMCs were stimulated with anti-CD2+anti-CD8 antibodies used at saturating concentrations [mAb 39C1.5 (rat IgG2a, anti-CD2) in combination with mAb 6F10.3 (mouse IgG1, anti-CD2); anti-CD28 mAbs (clone identification 28.2)] [89], [90]. Activation by anti-CD2+anti-CD28 monoclonal antibodies induces high-level, long-lasting and monocyte-independent proliferation of resting T cells, thymocytes and preactivated T cells [91], [92].

### Roche Amplicor quantitative assessments of HIV-1 RNA

One day after isolation, 6×10^6^ CD8^+^-depleted PBMCs or 2×10^6^ HLA DR^−^ CD4^+^ T cells were mock-treated or treated with different compounds. Six days after treatment, culture supernatants were tested for quantitative HIV-1 RNA levels by using the COBAS AmpliPrep/COBAS AMPLICOR HIV-1 MONITOR Test version 1.5, according to the manufacturer's instructions (Roche Diagnostics) (lowest detection limit: 50 copies HIV-1 RNA/ml of plasma).

### ChIP assays

ChIP assays were performed using the ChIP assay kit (EZ ChIP technology, Upstate). U1 cells were cross-linked after drug treatments. To detect chromosomal flanking regions, pellets were sonicated (Bioruptor sonicator) to obtain DNA fragments of 100–400 nt. Chromatin immunoprecipitations were performed with an antibody directed against RNAPII (catalog no. sc-899, Santa Cruz Biotechnology). To test aspecific binding to the beads, a purified IgG was used as a control for immunoprecipitation (catalog no. I-1000, Vector Laboratories). Quantitative real-time PCR reactions were performed using the MesaGreen qPCR mastermix (Eurogentec). Relative quantification using standard curve method was performed for each primer pair and 96-well Optical Reaction plates were read in an Applied Biosystems AbiPrism 7300 real-time PCR instrument (Absolute Quantification Method). Fold enrichments were calculated as percentages of input values normalized to an unrelated genomic region (18S rRNA DNA) and expressed as fold inductions relative to the values measured in mock-treated U1 cells. Primer sequences used for quantification in a region overlapping the LTR U5 region and the primer binding site region (to avoid interference with the 3'LTR region) (FW, 5′-TGGAAAATCTCTAGCAGTGGC-3′ and RV, 5′-GAGTCCTGCGTCGAGAGATCT-3′) and in the unrelated 18S rRNA DNA region (FW, 5′-TGGATACCGCAGCTAGGAATAA-3′ and RV, 5′-CCTCTTAATCATGGCCTCAGTTC-3′) were designed using the software Primer Express 2.0 (Applied Biosystems).

### RNA extractions and analyses of initiated and elongated HIV-1 transcripts

Total RNA samples were isolated using the RNeasy Plus kit (Qiagen) from 1×10^6^ U1 cells and digested with TURBO DNase (TURBO DNA-freeTM kit, Ambion). First strand cDNA was synthesized using SuperScript III Reverse Transcriptase (Invitrogen). Quantitative real-time PCR reactions were performed using the MesaGreen qPCR mastermix (Eurogentec). Initiated transcripts were detected with primers tar (FW, 5′-GTTAGACCAGATCTGAGCCT-3′ and RV, 5′-GTGGGTTCCCTAGTTAGCCA-3′). Elongated transcripts were detected with three different sets of primers: pol (FW, 5′-AATATGCAAGAATGAAGGGTGC-3′ and RV, 5′-TGCTTTCTGTGGCTATTTTTTGTA-3′), vif (FW, 5′-ATGGCAGGTGATGATTGTGTG-3′ and RV, 5′-CCATGTGTTAATCCTCATCCTGTC-3′) and nef (FW, 5′-GCCTGGCTAGAAGCACAAGA-3′ and RV, 5′-AGGTGTGACTGGAAAACCCAC-3′). β-actin mRNA copies were quantified with primers β-actin (FW, 5′-GTCGACAACGGCTCCGGC-3′ and RV, 5′-GGTGTGGTGCCAGATTTTCT-3′) specific for a 239 bp region in the β-actin mRNA. Quantitative real-time PCR reactions were performed for each primer pair and 96-well Optical Reaction plates were read in an Applied Biosystems StepOnePlus Real-Time PCR System (Comparative C_t_ (ΔΔC_t_) Quantification Method).

### Flow cytometry analyses

J-Lat 8.4 or 15.4 cells were mock-treated or treated for 24 h with the different compounds alone or in combination. Recoverded cells were washed twice in PBS, resuspended in PBS containing 4% paraformaldehyde and fixed for 1 h. Cells were next washed twice in PBS and resuspended in FACS buffer (PBS-BSA 0.1%-NaN_3_ 0.1%). The percentage of GFP-positive cells was measured on a CXP cytometer (Cytomics FC 500, Beckman Coulter) using CXP Software version 1.0 according to the manufacturer's instructions.

## Supporting Information

Figure S1 Dose-response curves of U1 cellular viability after HDACI treatment. U1 cells were mock-treated or treated with increasing concentrations of an HDACI belonging to one of the four structural families: short-chain fatty acids (A), benzamides (B), cyclic tetrapeptides (C), hydroxamic acids (D). At 24 h posttreatment, cellular viability was tested by measuring the mitochondrial dehydrogenase activity with the WST-1 reduction assay. The mock-treated value was arbitrarily set at a value of 100% of cellular viability. Each point is the mean from three separate experiments performed in triplicate. SE are intentionally not represented on the graph for clarity reasons.
(0.19 MB TIF)

Figure S2 Prostratin does not increase HDACI cytotoxicity in CD8+-depleted PBMC cultures from uninfected individuals. CD8+-depleted PBMCs were mock-treated or treated with VPA (2.5 mM), SAHA (2.5 µM), TSA (500 nM), NaBut (5 mM), MS-275 (5 µM), prostratin (5 µM) alone (A) or in combination (B). At 24 h posttreatment, cellular viability was tested by measuring the mitochondrial dehydrogenase activity with the WST-1 reduction assay. A value of 100% of cellular viability was arbitrarily assigned to the mock-treated value (A) or to the prostratin-treated value (B). Each value is the mean +/− SE from three separate experiments performed in triplicate.
(0.12 MB TIF)

Figure S3 Prostratin+HDACI cotreatment induces HIV-1 expression in a higher proportion of cells than the drugs alone. This figure shows as plots the same FACS results that are presented as histograms in Figure 3B in the manuscript. J-Lat 8.4 cells were mock-treated or treated with prostratin (5 µM), alone or in combination with different HDACIs [VPA (2.5 mM), SAHA (2.5 µM), TSA (500 nM), NaBut (5 mM) or MS-275 (5 µM)]. At 24 h posttreatment, cells were analyzed by FACS for GFP expression. The plots are representative of four independent experiments obtained with J-Lat 8.4 cells. Similar results were obtained with the J-Lat 15.4 T-cell clone (data not shown).
(0.18 MB TIF)

Figure S4 The prostratin+VPA cotreatment causes a more rapid and pronounced nucleosomal remodeling than the compounds alone. (A) Diagram indicating the positions of nucleosomes in the 5′ portion of the HIV-1 genome, the AflII and HinfI cutting sites and the probe used in indirect end-labeling. Bold, lower case letters are assigned to each HinfI cutting site (x, y and z) and are located next to the bands on the gel to permit their identification. (B) Nuclei were prepared from U1 cells mock-treated or treated with TNFα (10 ng/ml) (30 min), prostratin (5 µM), VPA (2.5 mM) and prostratin+VPA for 30 min, 1 h or 2 h and digested with HinfI. After DNA purification and in vitro restriction with PstI, DNA samples were analyzed by indirect end-labeling using probe A (93). Size markers (a, b, c, d, e, f, g) have been previously described (93).
(0.69 MB TIF)

Figure S5 The prostratin+VPA cotreatment does not induce levels of acetylated histone H4 higher than the levels observed after the treatments with VPA alone. Acetylated H4 levels in the nuc-1 region were assessed by ChIP experiments using U1 cells mock-treated or treated with prostratin (5 µM), VPA (2.5 mM) and prostratin+VPA for different periods of time. The proteins were cross-linked with formaldehyde for 10 min and DNA sheared. The cross-linked protein/DNA complexes were immunoprecipitated with an anti-Ac-H4 antibody. The protein/DNA cross-links were reversed and the purified DNA was amplified and quantified by real-time PCR using primers amplifying either the nuc-1 region or the vif region. Fold enrichments in the nuc-1 and vif regions were calculated as percentages of input values and expressed as fold inductions relative to the value measured with the nuc-1 primers in mock-treated U1 cells, which was arbitrarily set at a value of 1. Each value is the mean +/− SE from three separate experiments performed in duplicate.
(0.11 MB TIF)

Text S1 Supporting Information of Figure S1
(0.08 MB DOC)

Text S2 Supporting Information of Figure S2
(0.07 MB DOC)

Text S3 Supporting Information of Figure S5
(0.08 MB DOC)
