# Supplementary figures and images for: Synergistic Activation of HIV-1 Expression by Deacetylase Inhibitors and Prostratin: Implications for Treatment of Latent Infection
Source: PLoS One. 2009 Jun 30;4(6):e6093. doi: 10.1371/journal.pone.0006093 (PMC2699633; doi:10.1371/journal.pone.0006093)

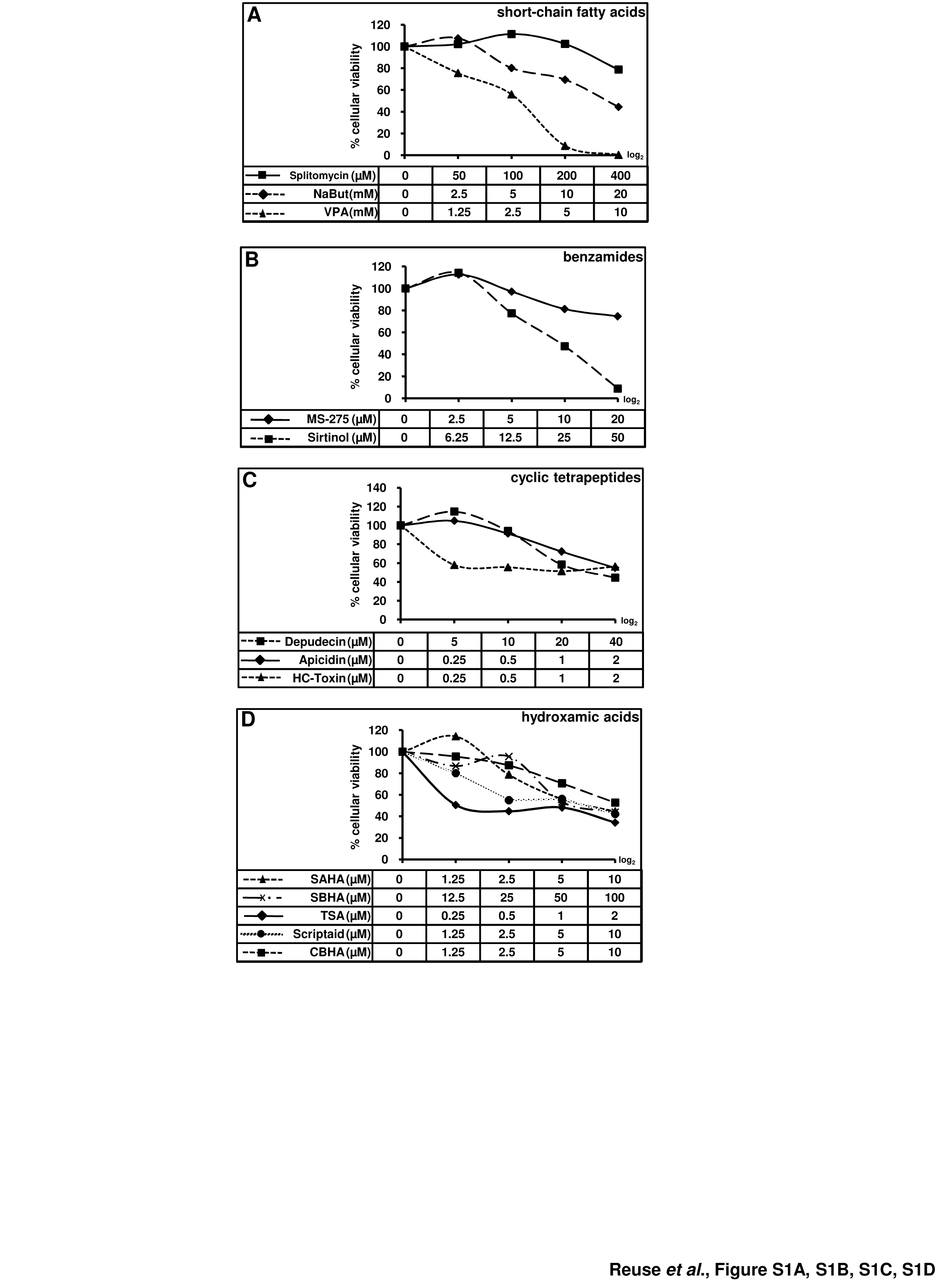

Supplement: Figure S1 — Dose-response curves of U1 cellular viability after HDACI treatment. U1 cells were mock-treated or treated with increasing concentrations of an HDACI belonging to one of the four structural families: short-chain fatty acids (A), benzamides (B), cyclic tetrapeptides (C), hydroxamic acids (D). At 24 h posttreatment, cellular viability was tested by measuring the mitochondrial dehydrogenase activity with the WST-1 reduction assay. The mock-treated value was arbitrarily set at a value of 100% of cellular viability. Each point is the mean from three separate experiments performed in triplicate. SE are intentionally not represented on the graph for clarity reasons. (0.19 MB TIF) [file pone.0006093.s001.tif]

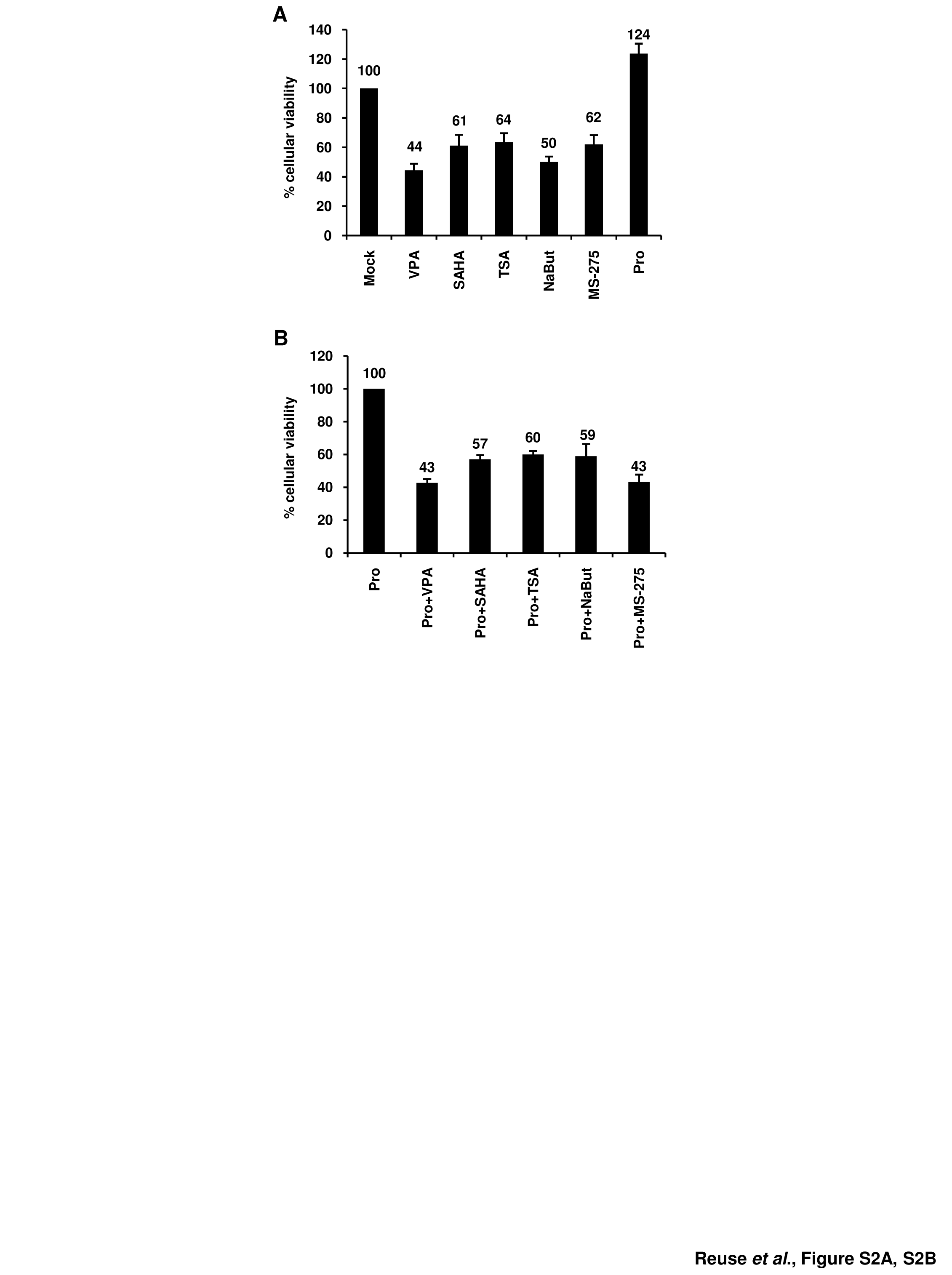

Supplement: Figure S2 — Prostratin does not increase HDACI cytotoxicity in CD8+-depleted PBMC cultures from uninfected individuals. CD8+-depleted PBMCs were mock-treated or treated with VPA (2.5 mM), SAHA (2.5 µM), TSA (500 nM), NaBut (5 mM), MS-275 (5 µM), prostratin (5 µM) alone (A) or in combination (B). At 24 h posttreatment, cellular viability was tested by measuring the mitochondrial dehydrogenase activity with the WST-1 reduction assay. A value of 100% of cellular viability was arbitrarily assigned to the mock-treated value (A) or to the prostratin-treated value (B). Each value is the mean +/− SE from three separate experiments performed in triplicate. (0.12 MB TIF) [file pone.0006093.s002.tif]

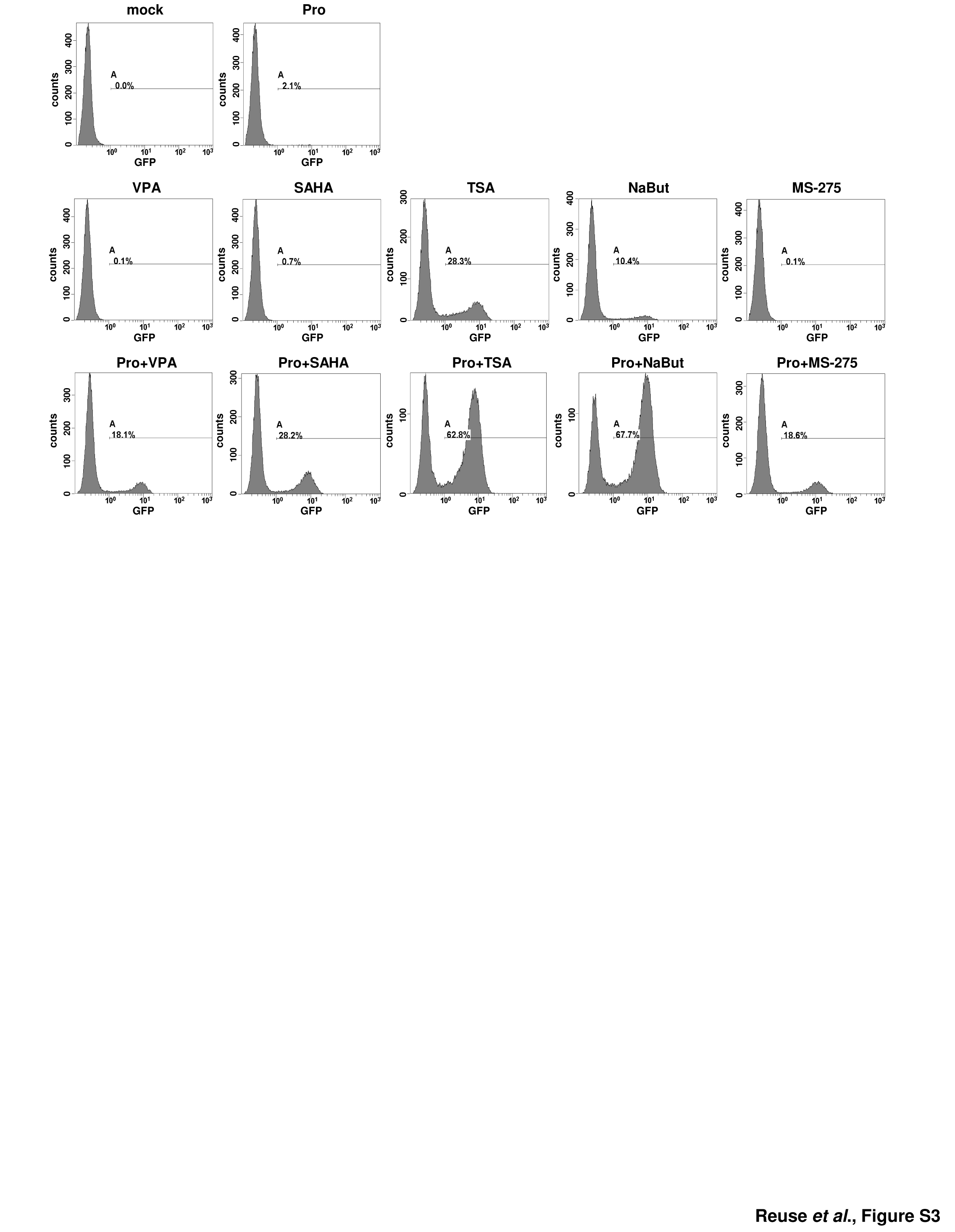

Supplement: Figure S3 — Prostratin+HDACI cotreatment induces HIV-1 expression in a higher proportion of cells than the drugs alone. This figure shows as plots the same FACS results that are presented as histograms in Figure 3B in the manuscript. J-Lat 8.4 cells were mock-treated or treated with prostratin (5 µM), alone or in combination with different HDACIs [VPA (2.5 mM), SAHA (2.5 µM), TSA (500 nM), NaBut (5 mM) or MS-275 (5 µM)]. At 24 h posttreatment, cells were analyzed by FACS for GFP expression. The plots are representative of four independent experiments obtained with J-Lat 8.4 cells. Similar results were obtained with the J-Lat 15.4 T-cell clone (data not shown). (0.18 MB TIF) [file pone.0006093.s003.tif]

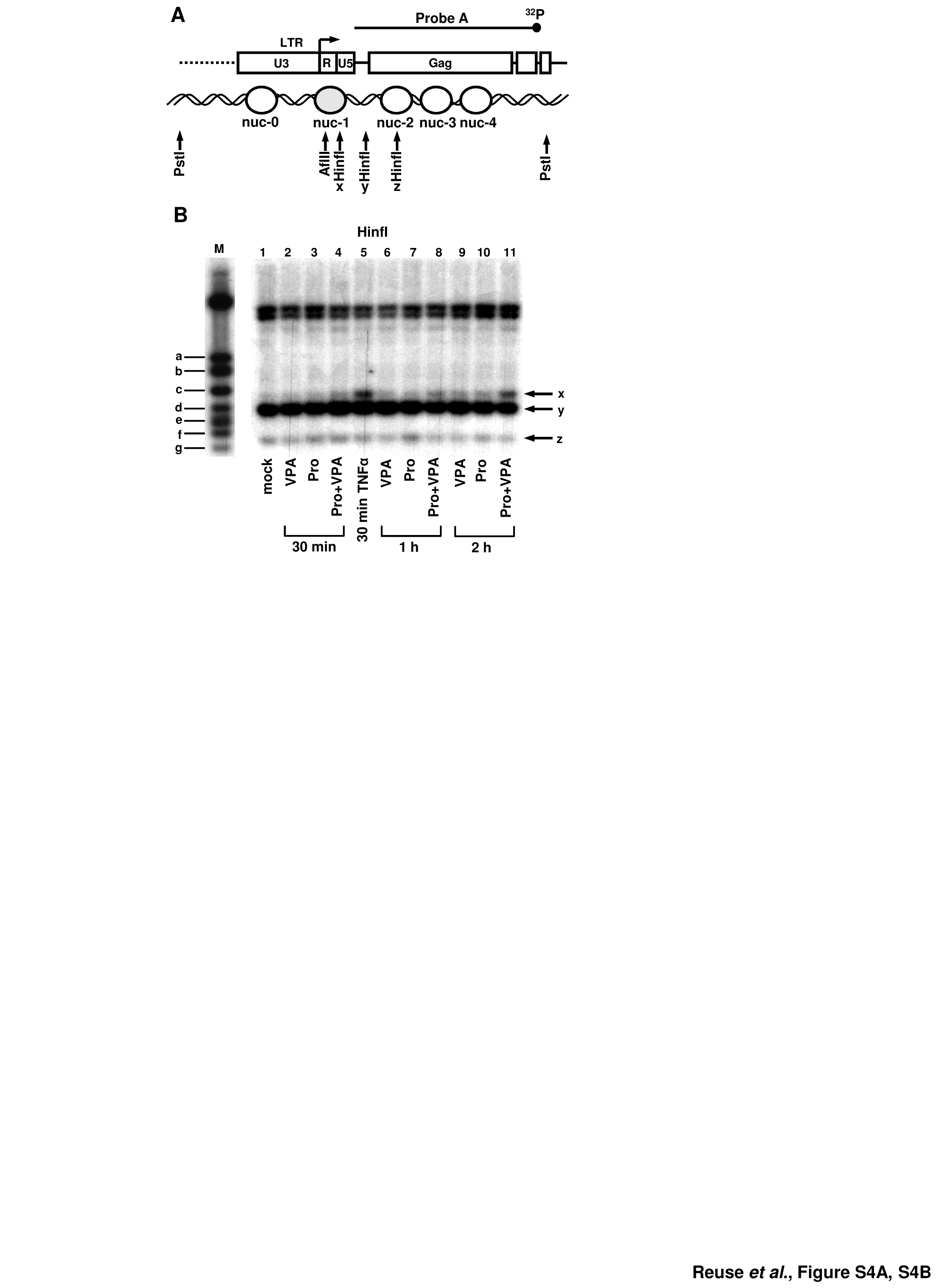

Supplement: Figure S4 — The prostratin+VPA cotreatment causes a more rapid and pronounced nucleosomal remodeling than the compounds alone. (A) Diagram indicating the positions of nucleosomes in the 5′ portion of the HIV-1 genome, the AflII and HinfI cutting sites and the probe used in indirect end-labeling. Bold, lower case letters are assigned to each HinfI cutting site (x, y and z) and are located next to the bands on the gel to permit their identification. (B) Nuclei were prepared from U1 cells mock-treated or treated with TNFα (10 ng/ml) (30 min), prostratin (5 µM), VPA (2.5 mM) and prostratin+VPA for 30 min, 1 h or 2 h and digested with HinfI. After DNA purification and in vitro restriction with PstI, DNA samples were analyzed by indirect end-labeling using probe A (93). Size markers (a, b, c, d, e, f, g) have been previously described (93). (0.69 MB TIF) [file pone.0006093.s004.tif]

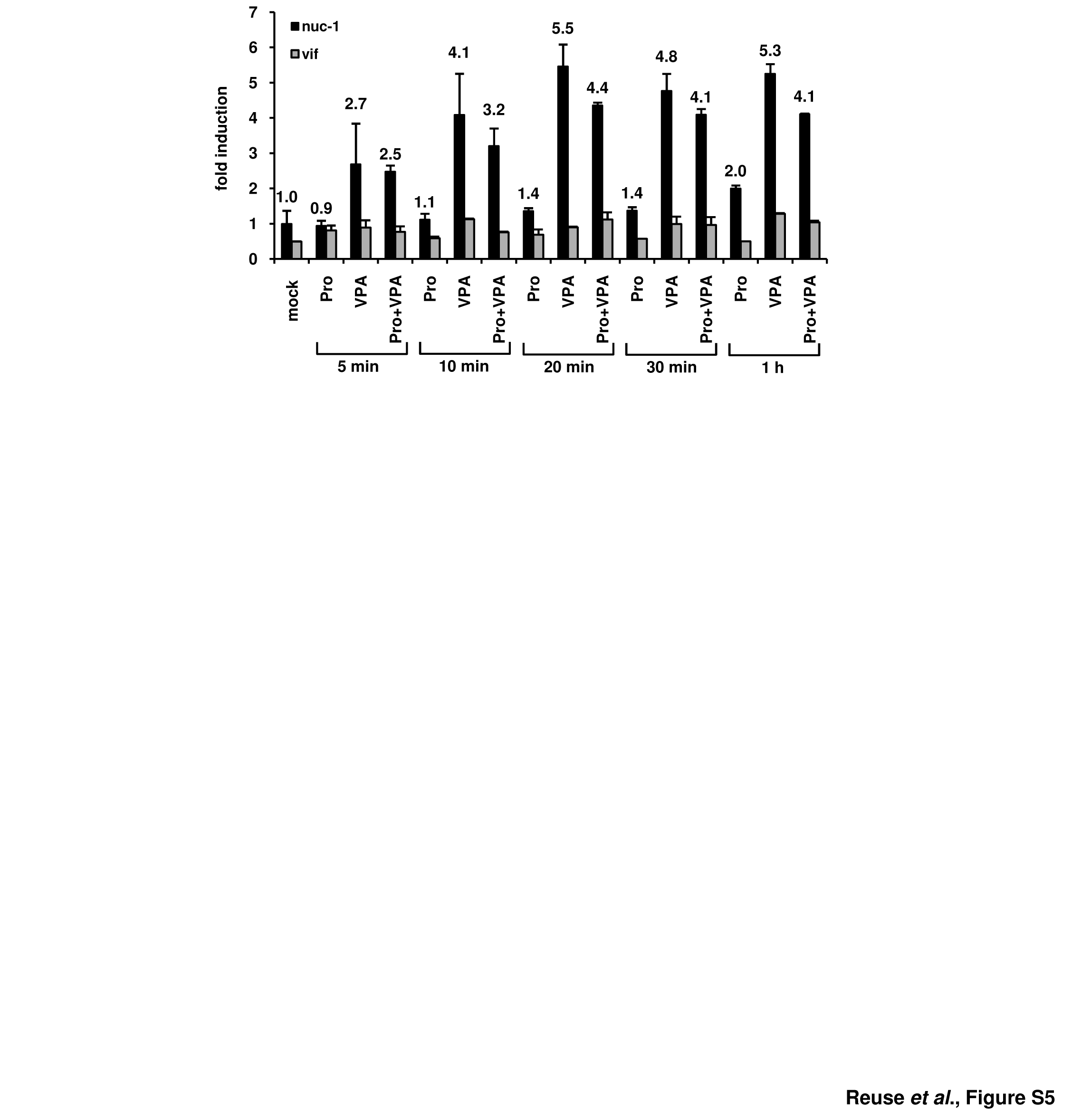

Supplement: Figure S5 — The prostratin+VPA cotreatment does not induce levels of acetylated histone H4 higher than the levels observed after the treatments with VPA alone. Acetylated H4 levels in the nuc-1 region were assessed by ChIP experiments using U1 cells mock-treated or treated with prostratin (5 µM), VPA (2.5 mM) and prostratin+VPA for different periods of time. The proteins were cross-linked with formaldehyde for 10 min and DNA sheared. The cross-linked protein/DNA complexes were immunoprecipitated with an anti-Ac-H4 antibody. The protein/DNA cross-links were reversed and the purified DNA was amplified and quantified by real-time PCR using primers amplifying either the nuc-1 region or the vif region. Fold enrichments in the nuc-1 and vif regions were calculated as percentages of input values and expressed as fold inductions relative to the value measured with the nuc-1 primers in mock-treated U1 cells, which was arbitrarily set at a value of 1. Each value is the mean +/− SE from three separate experiments performed in duplicate. (0.11 MB TIF) [file pone.0006093.s005.tif]
