# Supplementary material for: Synergistic Activation of HIV-1 Expression by Deacetylase Inhibitors and Prostratin: Implications for Treatment of Latent Infection
Source: PLoS One. 2009 Jun 30;4(6):e6093. doi: 10.1371/journal.pone.0006093 (PMC2699633; doi:10.1371/journal.pone.0006093)
Supplement: Text S1 — Supporting Information of Figure S1 (0.08 MB DOC) [file pone.0006093.s006.doc]

**TEXT S1**

**Figure S1** shows cytotoxicity dose-response analyses in the U1 cell line following treatment with various HDACIs belonging to the four structural groups.

**Figure S1 result.**

At the concentration at which NaBut or VPA exhibited the highest increase of viral production (5 mM or 2.5 mM, respectively) (Figure 1A), these short-chain fatty acid HDACIs were associated with a percentage of cell viability of 80% or 56%, respectively (Figure S1A). MS-275 exerted no cytotoxic activity on U1 cells (Figure S1B) at concentrations that induced HIV-1 production (2.5 and 5 µM) (Figure 1B) and still showed a weak cytotoxic effect at 20 µM (cell viability percentage of 75%) (Figure S1B). Depudecin, an epoxide HDACI, and Apicidin, a cyclic tetrapetide HDACI, provoked no significant cytotoxic activity upon U1 cells at 10 µM and 0.5 µM, respectively (Figure S1C). Interestingly, a high increase in viral production was observed at these concentrations (Figure 1C). After treatment with the cyclic tetrapeptide/epoxide HC-Toxin at 0.25 µM (the concentration for maximal viral reactivation, Figure 1C), the cell viability percentage was 60% and was stable up to 2 µM (Figure S1C). As shown in Figure S1D, U1 cell viability significantly decreased after a 24 h treatment with the hydroxamic HDACIs SAHA, SBHA (suberoyl bishydroxamic acid), TSA, Scriptaid and CBHA (M-carboxycinnamic acid bishydroxamide). At concentrations where we observed a strong viral reactivation (Figure 1D), percentages of cell viability were 96%, 44%, 55% and 87% in the presence of SBHA, TSA, Scriptaid and CBHA, respectively (Figure S1D). SAHA at 2.5 µM showed the highest viral reactivation potential (43-fold induction) and the lowest cytotoxicity (79% of cellular viability) (Figure 1D and Figure S1D).

**Figure S1 materials and methods.**

**WST-1 cytotoxicity assays.**

Cell viability was determined with a colorimetric assay, Cell Proliferation Reagent WST-1 (Roche Diagnostics). The test is based on the cleavage of the tetrazolium salt WST-1 in formazan by mitochondrial dehydrogenases in viable cells. The formazan dye was quantified by measuring the absorbance of the dye at 450 nm. 1x10^4^ U1 cells were treated for 24 h with various concentrations of the different compounds. The WST-1 reagent was next added and the absorbance was measured after 2 h at 37°C.
