# Supplementary material for: Synergistic Activation of HIV-1 Expression by Deacetylase Inhibitors and Prostratin: Implications for Treatment of Latent Infection
Source: PLoS One. 2009 Jun 30;4(6):e6093. doi: 10.1371/journal.pone.0006093 (PMC2699633; doi:10.1371/journal.pone.0006093)
Supplement: Text S2 — Supporting Information of Figure S2 (0.07 MB DOC) [file pone.0006093.s007.doc]

**TEXT S2**

**Figure S2** shows cytotoxicity analyses in human uninfected CD8^+^-depleted PBMCs following combined treatments with prostratin and clinically used HDACIs.

**Figure S2 materials and methods.**

**Isolation of CD8^+^-depleted PBMCs.**

CD8^+^-depleted PBMCs were isolated from buffy coats of healthy HIV-1-negative individuals (Blood Center, Charleroi, Belgium) by adding RosetteSep human CD8 depletion mixture (StemCell Technologies) to buffy coats before density centrifugation on a Ficoll-Hypaque gradient (Pharmacia)*.* Cells were washed with RPMI, resuspended at 2x10^6^ cells/ml of complete RPMI (RPMI, 10% fetal bovine serum, supplemented with 50 U/ml of penicillin, and 50 µg/ml of streptomycin).

**WST-1 cytotoxicity assays.**

Cell viability was determined with a colorimetric assay, Cell Proliferation Reagent WST-1 (Roche Diagnostics). The test is based on the cleavage of the tetrazolium salt WST-1 in formazan by mitochondrial dehydrogenases in viable cells. The formazan dye was quantified by measuring the absorbance of the dye at 450 nm. 2x10^5^ CD8^+^-depleted PBMCs were treated for 24 h with the different compounds. The WST-1 reagent was next added and the absorbance was measured after 2 h at 37°C.
