# Supplementary material for: Synergistic Activation of HIV-1 Expression by Deacetylase Inhibitors and Prostratin: Implications for Treatment of Latent Infection
Source: PLoS One. 2009 Jun 30;4(6):e6093. doi: 10.1371/journal.pone.0006093 (PMC2699633; doi:10.1371/journal.pone.0006093)
Supplement: Text S3 — Supporting Information of Figure S5 (0.08 MB DOC) [file pone.0006093.s008.doc]

**TEXT S3**

**Figure S5** shows the effects of the prostratin+VPA cotreatment for different time periods on the levels of acetylated histone H4 in the nuc-1 region.

**Figure S5 materials and methods.**

**Chromatin immunoprecipitation (ChIP) assays.**

The ChIP assays were performed as described in Flanagin et al., 2008. U1 cells were cross-linked after treatment with VPA and prostratin individually or in combination for different periods of time (5 min, 10 min, 20 min, 30 min and 1 h). To detect chromosomal flanking regions, pellets were sonicated (Bioruptor sonicator) to obtain DNA fragments of 100-400 nt. Chromatin immunoprecipitations were performed with an antibody directed against Ac-H4 (catalog no. 06-866, Upstate). To test aspecific binding to the beads, a purified IgG was used as a control for immunoprecipitation (catalog no. I-1000, Vector Laboratories). Quantitative real-time PCR reactions were performed using the MesaGreen qPCR mastermix (Eurogentec). Relative quantification using standard curve method was performed for each primer pair and 96-well Optical Reaction plates were read in an Applied Biosystems AbiPrism 7300 real-time PCR instrument (Absolute Quantification Method). Fold enrichments in the nuc-1 and *vif* regions were calculated as percentages of input values and expressed as fold inductions relative to the value measured with the nuc-1 primers in mock-treated U1 cells, which was arbitrarily set at a value of 1. Primer sequences used for quantification in the nuc-1 region (FW, 5’-CAGCTGCTTTTGCCTGTACTG-3’ and RV, 5’- TCCACACTGACTAAAAGGGTCTGA-3’) and in the *vif* region (FW, 5’-AGGAAAGCTAAGGACTGGTTTTATAGA-3’ and RV, 5’-CCTAGTGGGATGTGTACTTCTGAAC-3’) were designed using the software Primer Express 2.0 (Applied Biosystems).

**Figure S5 reference.**

Flanagin S, Nelson JD, Castner DG, Denisenko O, Bomsztyk K (2008) Microplate-based chromatin immunoprecipitation method, Matrix ChIP: a platform to study signaling of complex genomic events. Nucleic Acid Res 36(3).
